# Lineage tracing of T cell differentiation from T-iPSC by 2D feeder-free culture and 3D organoid culture

**DOI:** 10.3389/fimmu.2023.1303713

**Published:** 2023-12-15

**Authors:** Yoshitaka Ishiguro, Shoichi Iriguchi, Shinya Asano, Tokuyuki Shinohara, Sara Shiina, Suguru Arima, Yoshiaki Kassai, Yoshiharu Sakai, Kazutaka Obama, Shin Kaneko

**Affiliations:** ^1^ Shin Kaneko Laboratory, Department of Cell Growth and Differentiation, Kyoto University, Shogoin-Kawahara-cho, Sakyo-ku, Kyoto, Japan; ^2^ Takeda-CiRA Joint Program (T-CiRA), Fujisawa, Japan; ^3^ Department of Surgery, Graduate School of Medicine, Kyoto University, Shogoin-Kawahara-cho, Sakyo-ku, Kyoto, Japan; ^4^ Axcelead Drug Discovery Partners, Inc., Fujisawa, Japan; ^5^ T-CiRA Discovery and Innovation, Takeda Pharmaceutical Company, Fujisawa, Japan; ^6^ Department of Surgery, Osaka Red Cross Hospital, Fudegasaki-cho, Tennoji-ku, Osaka, Japan

**Keywords:** iPSC, T cell differentiation, CD4, 3D organoid, scRNA seq

## Abstract

**Introduction:**

T cells induced from induced pluripotent stem cells(iPSCs) derived from antigen-specific T cells (T-iPS-T cells) are an attractive tool for T cell immunotherapy. The induction of cytotoxic T-iPS-T cells is well established in feeder-free condition for the aim of off-the-shelf production, however, the induction of helper T-iPS-T cells remains challenging.

**Methods:**

We analyzed T-iPS-T cells matured in 3D organoid culture at different steps in the culture process at the single-cell level. T-iPS-T cell datasets were merged with an available human thymocyte dataset based in single-cell RNA sequencing (scRNA-seq). Particularly, we searched for genes crucial for generation CD4+ T-iPS-T cells by comparing T-iPS-T cells established in 2D feeder-free or 3D organoid culture.

**Results:**

The scRNA-seq data indicated that T-iPS-T cells are similar to T cells transitioning to human thymocytes, with SELENOW, GIMAP4, 7, SATB1, SALMF1, IL7R, SYTL2, S100A11, STAT1, IFITM1, LZTFL1 and SOX4 identified as candidate genes for the 2D feeder-free induction of CD4+ T-iPS-T cells.

**Discussion:**

This study provides single cell transcriptome datasets of iPS-T cells and leads to further analysis for CD4+ T cell generation from T-iPSCs.

## Introduction

T cell immunotherapy with functional T cells has shown great therapeutic potential for various types of neoplasms and infections. It has long been known that surgically-isolated tumor-infiltrating T cells have antitumor effects in melanoma (1–3). T -cell subsets have been studied in detail, with CD62L+ subsets receiving particular attention. These minimally differentiated T memory stem cells (T_SCM_), which have the stem-cell like ability to replicate and multipotently differentiate, mediate more potent antitumor responses than highly differentiated effector memory T cell (T_EM_) cells (4–6). T-cell engineering, such as chimeric antigen receptor (CAR), is another promising T-cell therapy approach. The CD19 antigen is a target for B cell malignancies in immunotherapies that have shown impressive results in patients with relapsed, chemorefractory B cell malignancies (7, 8).

However, neoplasms and chronic infections can evade T cell immunity by suppressing the expression of molecules recognized by T cells and thereby inhibit T cell activation. Furthermore, continuous exposure to the antigens causes an “exhausted” state, in which T cells lose their effective cytotoxic function and persistency (9, 10).

To expand the clinical application of T-cell immunotherapy, we and others have focused on induced pluripotent stem cells (iPSCs) as an alternative T-cell source, because iPSCs have higher replication capacity and pluripotency (11). We reported the regeneration of target antigen-specific CD8 ^+^ T cells from antigen-specific T cells reprogrammed into iPSCs (T-iPS-T cells) (12). T-iPS-T cells have a rearranged T cell receptor (TCR) gene in the genome of the original antigen-specific CD8 ^+^ T cells and show antigen-specific monoclonality in the TCR expression. Importantly, T-iPS-T cells have higher proliferative activity and less of the exhausted phenotype than the original T cells. We previously reported the generation of feeder-free cytotoxic T-iPS-T cells and their effectiveness for “off-the-shelf” production (13).

Helper T cells are additional candidates for T cell immunotherapy. In CAR-transduced T cells, primary CD8 ^+^ T cells in combination with CD4 ^+^ T cells showed superior antitumor reactivity *in vivo* (14–16). However, the generation of helper T-iPS-T cells in feeder-free conditions has not been reported. Recent studies have demonstrated that 3D organoids provide a good environment to differentiate CD4^+^ T cells from hematopoeitc stem/progenitor cells and iPSC-derived endomesodermal progenitors (17–20).

**Figure 1:**
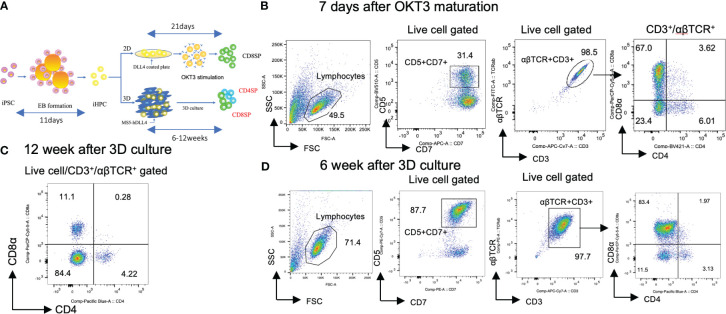
Generation of CD4SP and CD8SP T-iPS-T cells by organoid culture. **(A)** Schemes of CD4 SP and CD8SP T-iPS-T cell differentiation. EBs were generated from a single-cell dissociated T-iPSC proliferated in feeder- and serum-free condition. The emerging iHPCs were differentiated on 2D and 3D conditions. The T cell maturation in the 2D condition required OKT3 stimulation, however, no additional stimulation is introduced in the 3D condition. **(B)** Flow cytometry analysis of T-iPS-T cells differentiated under 7 days OKT3 stimulation based on the expression levels of CD4 and CD8a. Results represent 3 independent experiments. **(C)** Flow cytometry analysis of hematopoietic differentiation from a T-iPSC clone, TkT3V1-7, based on the expression levels of CD4 and CD8a at 12 weeks after ATO induction showing clearly independent CD4^+^CD8^-^ and CD4^-^CD8^+^ populations. The gating strategies involved the delineation of live cells expressing CD3 and abTCR. Results represent more than 3 independent experiments. **(D)** Flow cytometric analysis of T-iPS-T cells on organoid culture at 6 weeks based on CD4 and CD8a expressions. Distinct CD4^+^CD8^-^ and CD4^-^CD8^+^ populations were already emerging in this 6 weeks culture. Results represent more than 3 independent experiments.

Therefore, in this study, we molecularly characterized reprogrammed T cells (T-iPSCs) during their differentiation into CD4+ T-iPS-T cells in 2D feeder-free culture (2D culture) and 3D organoid culture (3D culture). We acquired and analyzed single cell transcriptome data of T-iPS-T cells in the two culture conditions (**Figure 1A**) and found the gene expression profiles were similar to human primary thymocytes during the maturation process. Additionally, a certain population from the 2D condition was included in the helper T cell cluster even though this population did not express distinctive helper markers such as CD4. These findings provide further evidence that T-iPSCs have potential for the production of “off-the-shelf” helper T-iPS-T cells.

## Results

### Matured CD4SP T cells were induced from iHPCs by organoid culture

Hematopoietic progenitor cells derived from T-iPSCs (iHPCs) were obtained using a modified embryo body (EB) formation protocol (13). The differentiated EB contained over 50% of CD34^+^CD43^+^ iHPCs (**Figure S1A**). The obtained iHPCs were differentiated to CD4^+^CD8^+^ αβTCR^+^ double-positive cells (DP cells) on DLL4-coated plates under 2D feeder-free culture (2D culture) (**Figure S1B**). Subsequently, the DP cells were induced to differentiate into mature CD4^-^CD8αβ^+^ (CD8SP) T-iPS-T cells by TCR stimulation, as previously described (21). However, this 2D culture failed to induce a CD4^+^CD8^-^ (CD4SP) population after TCR signal activation by anti-CD3 antibody (clone OKT3) in monitored 7 days (**Figure 1B**). CD8α and CD8β exhibited proportional expression (**Figure S2A**).

We next tested whether organoid-culture T-cell differentiation induces CD4SP subsets from the same iHPCs used in the 2D culture. To this end, we adopted an artificial thymic organoid (ATO) system (17). The frequency of cord blood-derived CD3^+^αβTCR^+^ T cells in ATOs has been reported to be highly consistent across experiments and includes CD8SP and, to a lesser extent, CD4SP T cells. The ATO system also succeeded to induce T cell lineage differentiation in the case of endomesodermal progenitors derived from iPSCs (iEMPs) (18). Our iHPCs were able to generate organoid-like structures in a similar manner when mixed with a murine stromal cell line, MS5, expressing a NOTCH ligand, DLL4, which resulted in better outcomes in the number of differentiated cells than DLL1 (data not shown), and cultured in air-liquid phase cultures and differentiated into T-iPS-T cells including an CD4SP population by 12 weeks (**Figure 1C**). The DP and each SP subset emerged as early as 6 weeks in ATO (**Figure 1D**). CD8α and CD8β exhibited proportional expression (**Figure S2B**).

These findings suggest that iHPCs have intrinsic CD4+ cell differentiation potential but require additional signals unique to organoid cultures when compared with 2D cultures for CD4SP T cell generation.

### CD4SP and CD8SP appeared after 4 weeks of organoid culture differentiation

**Figure 2:**
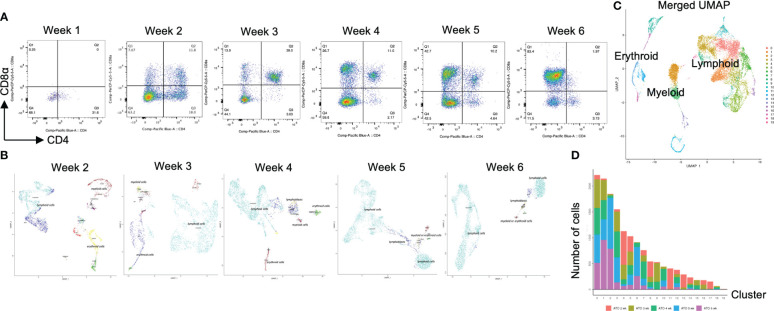
scRNA-seq analysis of T-iPS-T cells in organoid culture. **(A)** Flow cytometry plots of organoid cultures showing CD4 and CD8a expressions showing gradual maturation process of T cells. The maturation initiated from presenting CD4^+^CD8^-^ population. The population gradually disappeared by 3 weeks and became CD4^+^CD8^+^. The CD4^+^CD8^-^ population emerged again by 6 weeks. The gating strategies involved the delineation of live cells expressing CD3 and abTCR. Results represent more than 3 independent experiments. **(B)** Weekly UMAP plots with RCA analysis of 3D organoid cultures. At week 2, differentiating cells were heterogeneous and included erythroid, myeloid and lymphoid cells. However, as the culture progressed, the cellular heterogeneity decreased, and a large fraction of cells was assigned to lymphoids or lymphoblasts by week 6. **(C)** UMAP plot of cumulative data across all samples including all time points of organoid samples color-coded by clusters. This merged plot contained erythroid, myeloid, stem-like clusters other than lymphoid cluster. The clusters were named depending on the RCA score. **(D)** Histograms showing the number of cells in each week. Cluster 0, 1, 2 (highlighted with red) belonged to lymphoid cells and 6 week sample contained mainly lymphoid cells. Most of the non-lymphoid clusters belonged to samples from earlier differentiation cultures.

The above results prompted us to explore molecular differences between the organoid and 2D cultures to identify potential candidates to generate CD4^+^ T-iPS-T cells. In the human thymus, thymus-seeding progenitors differentiate sequentially into progenitor T cells, immature single-positive (ISP) cells expressing CD4, and DP T cells, which finally mature into CD4SP or CD8SP T cells (22). We therefore performed a weekly flow cytometric analysis of differentiating cells within the organoid cultures and found iHPCs showed a similar flow cytometry profile (**Figure 2A**). These results were representative and supplementary dataset results were archived in **Figure S3**. The cellular quantity was modest in the 1 week sample due to the persistent minority status of the CD3+αβTCR+ population (**Figure S4**). CD4^-^CD8^-^ (DN) cells were remaining in the dominant population at 1 week and transient ISP cells was appeared at 2 weeks. Similar to 2D culture (23), iHPCs derived from T-iPSCs also gave rise to DP cells as early as 2 weeks after the initiation of the organoid culture. The frequency of DP cells steadily declined as the culture proceeded and disappeared by 12 weeks (**Figure 1C**). Unlike 2D cultures, mature T cells appeared at 4 weeks without additional TCR stimulation. Based on these findings, we hypothesized that the maturation of T-iPS-T cells after the iHPC stage occurred at 2 to 6 weeks. Samples collected from each week contained cells at various differentiation stages. For a precise single-cell RNA sequencing (scRNA-seq) analysis of each step, samples from organoid culture at 2 to 6 weeks were collected separately and analyzed in an integrated manner.

### Transcriptomic profile of iHSC-derived T-iPS-T cells showed the rational transitional of T cells along with the expression of Cite-seq

**Figure 3:**
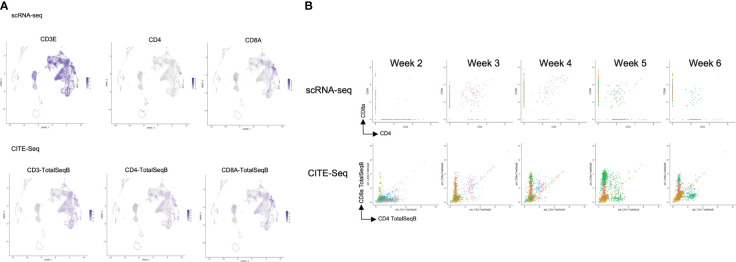
Comparison of the scRNA-seq and the CITE-seq analysis of T-iPS-T cells in organoid culture. **(A)** Scaled expressions of CD3e, CD4 and CD8A mRNA (top) and these surface proteins, labelled with oligonucleotide-labeled antibodies, (bottom) of the merged dataset shown on UMAP plots generated as in **Figure 2C**. CD3e was presented most of the lymphoid population. CD4 and CD8 were partially presented in lymphoid population in this analysis, however, on the cite-seq analysis, CD3, CD4 and CD8A were presented on the surface of the cells of this lymphoid cluster. **(B)** mRNA expression (top) of ATO cultures compared with CITE-seq analysis (bottom), showing CD4 and CD8A expression levels. The CITE-seq analysis showed similar tendency to FACS results (**Figure 2A**).

**Figure 4:**
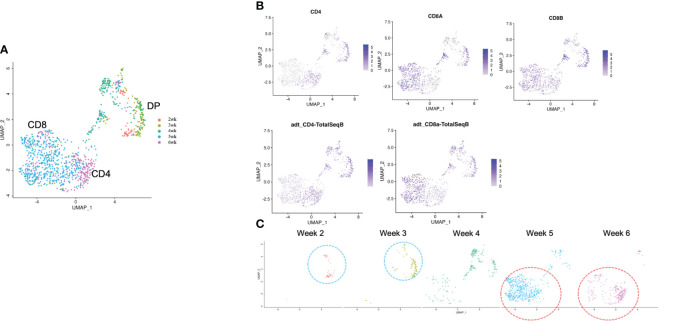
scRNA-seq analysis of extracted T cell lineage-committed population of the 3D organoid culture T-iPS-T cells. **(A)** UMAP plot of selected organoid-cultured cells committed to T cell lineage based on CITE-seq results color-coded by cultured week. **(B)** UMAPs of the gene expressions of CD4, CD8A, and CD8B (top) and protein surface expressions of CD4 and CD8A through CITE-seq(bottom). **(C)** UMAP plots as in **(A)** depicted with individual weeks. The DP population was highlighted with blue circle and the SP population was highlighted with red circle.

After computational analysis, each sample was divided into 9 to 14 clusters individually. As weeks went by, the number of clusters tended to decrease (**Figure S5A**). In order to assign cell types without bias to each cluster comprising each sample, we subjected the data to a global panel of the reference component analysis (RCA) (24) to classify samples into major groups (**Figures 2B**; **S5B**). At week 2, differentiating cells were heterogeneous and included erythroid, myeloid and lymphoid cells (**Figure 2B**, left panel). However, as the culture progressed, the cellular heterogeneity decreased, and a large fraction of cells was assigned to lymphoid or lymphoblast clusters by week 6 (**Figure 2B**, right panel). Consistent with these findings, the merged plot also contained erythroid, myeloid, stem-like and other clusters (**Figure 2C**; **S5C**), but most of these non-lymphoid clusters belonged to samples from earlier differentiation cultures (**Figures 2D**). An expression analysis of *CD3E*, *CD4*, and *CD8A* showed that the mRNA expression levels of *CD4* and *CD8A* were too low to clearly segregate the cells into DN, ISP, DP, CD4SP and CD8SP (**Figure 3A**, top row). We therefore simultaneously implemented a CITE-seq analysis (**Figure 3A**, bottom row), which is a method to analyze cellular surface proteins by labeling the cells with oligonucleotide-labeled antibodies (25). The CITE-seq analysis of the CD4 and CD8A plots indicated a similar transition (**Figure 3B**, bottom row) to that seen in **Figure 2A**. The analysis confirmed the scRNA-seq data correctly included the target cell population (**Figure 3B**, top row). For a more precise analysis, T cell lineage-committed populations were extracted from the original datasets based on the CD4 and CD8A surface expression obtained by the CITE-seq results (**Figure 4A**). Using this extraction, we were able to obtain a dataset where CD4 and CD8A feature plots showed DP and SP populations (**Figure 4B**). UMAP plots indicated that the DP population was dominant at 2 and 3 weeks, but the main population gradually transitioned to the SP population at 4 to 6 weeks (**Figure 4C**), but the main population gradually transitioned to the SP population at 4 to 6 weeks (**Figure 4D**). The results were then validated by transcription markers. RAG1 and RAG2 expression was skewed to the DP phase, ZBTB7B expression was relatively specific to the CD4 expression, and BCL11B, GATA3 and RUNX3 were widely expressed (**Figure S5D**).

### Single-cell transcriptome similarities in T-iPS-T cells and primary thymocytes differentiation

**Figure 5:**
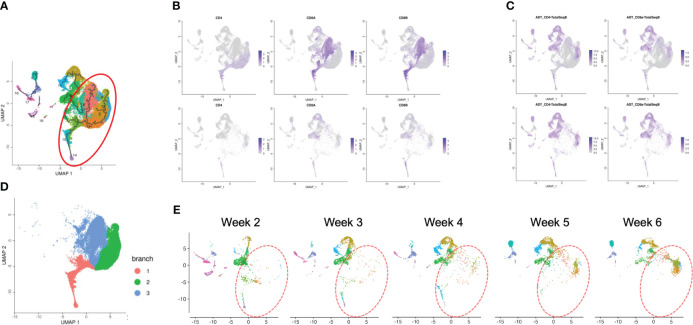
Pseudotime analysis of a merged plot of organoid culture T-iPS-T cells and thymocytes. **(A)** Trajectory analysis of all organoid cultured T-iPS-T cell data merged with published human thymocyte datasets. The red circle was T cell lineage cells depending on CD4, CD8A and CD8B expression of thymocyte datasets. **(B)** Feature plots of CD4, CD8A and CD8B in merged dataset of 3D organoid cultured T-iPS-T data and published human thymocyte datasets (top) and in 3D organoid cultured T-iPS-T data (bottom). **(C)** Feature plots of CD4 and CD8A through CITE-seq. The results were showed on merged plots (top). The human thymocyte datasets were subtracted in bottom figures. **(D)** T cell lineage cells which highlighted with red circle in **(A)**. The trajectory branches were divided with color. (immature cells (red), CD4SP (green), CD8SP (blue)). **(E)** Distinct UMAP plots of T-iPS-T cells shown by week projected onto UMAP generated as in **(A)**. T-iPS-T cells at 2 weeks mainly projected onto DP clusters defined by human thymocytes and merged onto SP clusters as the differentiation progressed.

To confirm how T-iPS-T cell differentiation approximates that of human thymocyte differentiation, we compared T-iPS-T cell and thymocyte gene expression profiles. Human thymocyte datasets were obtained from a published database which contained human infant thymocytes obtained from 5 donors (26). A combined trajectory analysis of the human thymocyte datasets and organoid culture data (**Figure 2C**) is shown in **Figure 5A**. In the feature plot of merged data, CD4, CD8A and CD8B expressions were featured in **Figure 5B**. As the expression level was lower in organoid culture data, we also confirmed these markers with CITE-seq results (**Figure 5C**). Depending on these results, we selected right side clusters highlighted with red circle in **Figure 5A** as T cell related clusters. The clusters specific to the organoid culture were omitted from the merged dataset. The trajectory branches divided the cells into immature (red), CD4SP lineage (green) and CD8SP lineage (blue) based on the gene expression of human thymocyte datasets (**Figure 5D**). This observation implied the CD4SP and CD8SP populations were derived from the DP population. The mixed datasets in **Figure 5A** comprised mainly human thymocyte data, indicating that the trajectory analysis successfully recapitulated T cell differentiation in silico. T-iPSCs contained in the red circle at 2 weeks mainly projected onto DP clusters (branch 1) defined by human thymocytes and merged onto SP clusters (branch 2 or 3) as the differentiation progressed (**Figure 5E**). A scRNA-seq analysis of the organoid-cultured T-iPSCs and published human thymocyte datasets indicated that the gene expression profiles of differentiating T-iPS-T cells were transcriptomically similar to human thymocytes.

### Pseudotime analysis identified gene candidates for the differential transition to CD4SP cells in organoid culture

**Figure 6:**
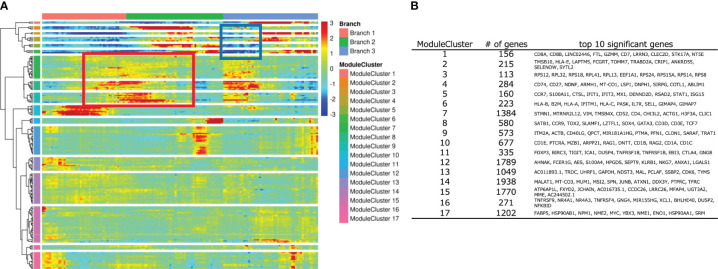
Analysis of developing T-iPS-T with human thymocytes along with trajectory. **(A)** Heatmap of DEG modules along the pseudotime. **(B)** Top 10 significant genes of each module cluster.

To identify key genes in the CD4SP T-iPS-T differentiation from DP cells, we performed a pseudotime analysis on the merged datasets of organoid cultured cells and thymocytes contained in 3 branches (**Figure 6A**). Significantly regulated genes were further subjected to a hierarchical clustering analysis, which yielded 17 modules. The top 10 genes in each module cluster were shown in **Figure 6B**. To investigate differentially expressed genes during DP to CD4SP T cell commitment, we focused on module clusters #7, #8, and #9 (**Figure 6A**; boxed in red rectangular), which contained genes that were upregulated between the immature and CD4SP lineage stages, and on module clusters #2, #5, and #6 (**Figure 6A**; boxed in blue rectangular), which contained genes that were downregulated during the relatively initial phase of CD8SP lineage commitment. We hypothesized that genes included in these module clusters were candidate genes for the transition from the DP phase to CD4SP T cells.

### 2D differentiation samples include a primed helper population

To identify potential genes/pathways for CD4SP T cell differentiation by feeder-free culture, we compared single-cell transcriptomes of cells derived from the 2D culture (CD8SP only) and the organoid culture (CD4SP and CD8SP cells). We first performed a quality analysis of the single-cell transcriptome of terminally differentiated cells in 2D culture (**Figure 1B**) and annotated them on the basis of marker gene expressions in each cluster projected onto the UMAP plot (**Figures S6A**). We analyzed datasets collected after 6 weeks of differentiation in organoid culture (**Figure 2B**) for minor clusters with features resembling myeloid and erythroid phenotypes. The combined UMAP plots of these two datasets showed unique clusters in the organoid culture (**Figures S6B, C**). Cluster 3 contained GZMK (27), CD69 and ITGAL (28) (29), which were reported to be up-regulated in lymphocytes (**Figure S6D**). These genes were also up-regulated in cluster 4, which was another organoid culture-specific cluster.

**Figure 7:**
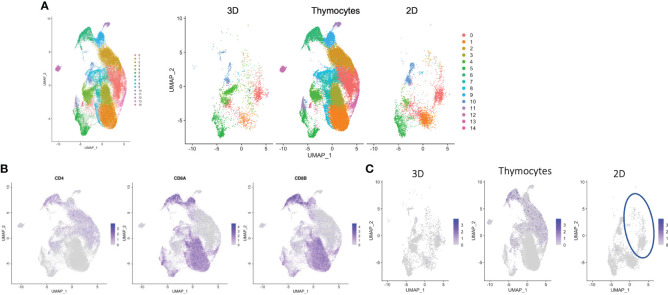
Transcriptomic profiles of differentiated cells from 2D and organoid culture by scRNA-seq analysis. **(A)** Merged UMAP of organoid-cultured cells, 2D-cultured cells and published human thymocytes. **(B)** Scaled expressions of CD4, CD8A and CD8B on UMAP generated as in **(A)**. **(C)** Scaled expression of CD4 in organoid-cultured cells, thymocytes and 2D-cultured cells. The blue circle shows the population presented with CD4^+^ SP cells in thymocytes and organoid-cultured cells.

Datasets for organoid culture at 6 weeks, 2D culture and thymocytes were merged and processed as a UMAP plot (**Figure 7A**, left). Unmerged datasets also showed shared clusters (**Figure 7A**, right). Thymocytes showed a variety of clusters. Cluster #1 and #3 showed dominantly up-regulated CD8 expression, but clusters #0 and #2 showed CD4 expression. These cluster gene expressions were higher in the thymocyte datasets than the culture data. The 2D and organoid culture clusters indicated T cell-related gene expressions (**Figure S7A**). Unexpectedly, some cells from the 2D culture samples also projected onto the cluster (**Figure 7C**, circled) for CD4 expression in the thymocyte and organoid culture data (**Figures 7B, C**), which disagrees with the FACS analysis in **Figure 1B**. *SELL* (30), which is related to Tscm, was expressed in cluster #0, and *BACH2* (31), which is related to T cells, was expressed in cluster #2 (**Table S1**). These results indicate that some cells in 2D culture have helper function.

To elucidate the mechanisms for the low CD4 expression in 2D culture, we compared the gene expression dynamics during the transition to mature CD4SP T cells. We utilized the regulated gene sets identified by the pseudotime analysis (**Figure 6A**) by evaluating the expression of the top 10 genes in module clusters #2, #5, #6, #7, #8, #9 in the 2D culture dataset, assuming that their differential expression was related to CD4 expression in organoid culture. For the potent helper functional clusters (**Figure 7C**, circled), *SELENOW* and *SYTL2* in module cluster #2, *S100A11* and *STAT1* in module cluster #5, *GIMAP4*, *GIMAP7*, *IFITM1*, and *IL7R* in module cluster #6, and *LZTFL1*, *SATB1*, *SLAMF1*, and *SOX4* in module cluster #8 showed higher expression levels in organoid culture than 2D culture (**Figure S7B**).

## Discussion

Adoptive immunotherapy with antigen-specific cytotoxic T-iPS-T cells are reported to have antitumor reactivity (32, 33). However, the generation of T-iPS-T cells that have helper function has not been achieved. In this study, we performed a time-course analysis of differentiating T cells from organoid culture by means of flow cytometry, scRNA-seq, and CITE-seq to elucidate molecular clues that are associated with the transition from DP T cells to CD4SP T cells in 2D feeder-free conditions. A computational analysis revealed that T-iPSCs differentiated to T cells in organoid culture have transcriptional similarity to human thymocytes. In addition, our study identified key genes, including genes previously known to play pivotal roles in CD4 helper T cell differentiation and novel genes regulating CD4 helper T-cell genesis, in the organoid culture.

Previous CD4SP T cell induction from iPSCs in organoid culture passed an iEMP stage (18), but it remains unknown whether organoids were required to induce CD4SP T cells. The same group also induced hematopoietic stem/progenitor cells from cord blood and peripheral blood CD34+ cells into CD4SP T cells using organoids. Our iHPCs derived from EBs could differentiate into CD4 SP T cells as well. This result implied this organoid culture is applicable to cells at another differentiation stages than iEMP stage and showed that organoid culture is sufficient for CD4SP T-cell induction after the iHPC stage.

We have reported an induction method for CD8SP T cells passing from iHPCs through DP cells (13). The method can be separated into two sections comprising of NOTCH-delta ligand stimulation followed by CD3 stimulation. However, in organoid culture, iHPCs are cultured only in the presence of NOTCH ligand without additional CD3 stimulation. These results may indicate *in vitro* CD4^+^ T cell differentiation does not require TCR signaling and that some soluble factors such as cytokines and/or cell-to-cell interactions other than TCR-MHC may play a dominant role. Alternatively, each cell in the aggregates of organoid culture may provide TCR signaling in the form of TCR-MHC interactions either from human iPSCs or murine MS5 after gene editing. Assessments with HLA knock-out iPSC lines will provide further evidence.

In this study, we analyzed the T cell induction of iHPCs in 2D and organoid cultures. We compared the cell products with a single-cell human thymocyte transcriptomic dataset (26). Surface marker dynamics during the T-iPS-T cell maturation in organoid culture was consistent with that of primary thymocytes. These findings support our notion that an analysis of differentially expressed genes in T-iPSCs with the same genetic background in 2D and organoid cultures can guide the generation of CD4^+^ T-iPS-T cells in feeder-free condition. Unexpectedly, we found that some cells in 2D culture had already acquired some molecular features of CD4SP T cells even though flow cytometry showed they did not express CD4. This result implied some T-iPS-T cells may contain CD4 primed T cells that fail to fully commit to mature CD4SP T cells. Accordingly, we focused on master regulators for CD4SP T cell maturation and found that Zbtb7b and CD40LG expression was slightly elevated in 2D culture, although the expression levels of these transcription factors were low in the thymocyte samples, making it difficult to compare the expression levels among the three cell groups (2D culture, organoid culture, primary thymocytes).

Bcl11b is an important transcription factor for T cell differentiation in multiple steps (34). In the early stage of mouse T cell differentiation, DN phenotypes are divided into four stages (DN1 to DN4). Bcl11b is first expressed in the DN2 stage to promote T cell commitment and prevent alternative lineages (35). Additionally, Bcl11b is indispensable for T cell positive selection and the divergent development to SP T cells. For more effective helper T cell induction, Bcl11b is needed to repress Runx3, which directly inhibits Zbtb7. It also acts as a positive regulator of Zbtb7b (36). In the present study, Bcl11b and Runx3 were expressed normally but Zbtb7b negligibly in a helper cluster candidate in 2D culture (**Figure S7A**). We therefore suppose Bcl11b is dysregulated in this cluster such that it does not repress Runx3 or agonize Zbtb7b sufficiently, which might cause CD4 de-expression. It implies a possibility that Bcl11b enforced expression on T-iPSCs may cause the promotion of CD4SP differentiation.

We also compared gene expressions by a pseudotime analysis of organoid cultured cells and thymocytes; these analyzed genes had relatively low expression in 2D culture. Among them, SELENOW (37), *Gimap 4*, and *7* (38), SATB1 (39), SALMF1 (40), SYTL2, S100A11, STAT1, IFITM1 (41) and IL7R (42) were previously reported as possible factors for T cell differentiation. However, LZTFL1 and SOX4 were not previously described as essential factors for DP to SP differentiation. We will confirm the role of these gene candidates with genetic engineered T-iPSCs. We will also consider using CRISPR Cas9 screening library. In summary, we generated CD4SP T-cell generation from iHPCs derived from iPSCs along with the ATO protocol (17), showed T-iPS-T cells in organoid culture are transcriptomically similar to human thymocytes and found gene candidates that may promote sustainable CD4SP and CD8SP T-iPS-T production in feeder-free conditions.

## Methods

### Human induced pluripotent stem cell lines

The human iPSC line TKT3V1-7 was induced from an antigen-unspecific CD3 T cell of a healthy volunteer by using a retroviral vector harboring OCT3/4, KLF-4, SOX-2, and C-MYC (12).

### Cell lines

To generate MS5-hDLL4, MS5 cells (43) were transduced with a retroviral vector encoding full-length human *DLL4*. The highest 5% of DLL4-expressing cells were sorted by FACS using an anti-DLL4 antibody and passaged in DMEM/10% fetal calf serum (FCS). Stable expression was confirmed by flow cytometry for DLL4 expression after several weeks of culture.

### Flow cytometry and antibodies

The following conjugated antibodies were used for flow cytometry staining: CD3 (UCHT1), CD3e (UCHT1), CD4 (OKT4), CD5 (UCHT2), CD7 (CD7-6B7), CD8a (SK1), CD8b (S1D18BEE), CD34 (4H11), CD43 (1G10), and TCRab (WT31). The antibodies were purchased from BD Biosciences (San Joe, CA, USA), Beckman Coulter (Marseille, France), BioLegend (San Diego, CA, USA), eBioscience (San Diego, CA, USA), abcam (Cambridge, UK) and R&D Systems. The stained cell samples were analyzed using a FACS Aria II flow cytometer (BD Biosciences), and the data were processed using FlowJo software (Tree Star, Ashland, OR). The staining incubation was performed in FACS buffer (2% FBS in D-PBS) for 30 min on ice.

### Generation of iHPCs from iPSCs

We previously reported a method for the differentiation of iPSCs into T cells (12), which can be obtained at Protocol Exchange. iPSCs were expanded for 6–7 days on iMatrix-511 in StemFit AK02N and dissociated into single cells using 0.5× TryPLE select (Thermo Fisher Scientific). Whole 3-6 × 10^5^ cells were resuspended in StemFit AK02N supplemented with 10 μM Y-27632 (FujiFilm Wako) and 10 μM CHIR99021 (Tocirs Bioscience) and cultured in 6-well ultra-low attachment plates (Corning) for 24 h. Subsequently, the EBs were collected, settled down to the bottom of the tube, and resuspended with 2 mL StemPro-34 (Thermo Fisher Scientific) supplemented with 10 ng/mL penicillin/streptomycin (Sigma), 2 mM Glutamax (Thermo Fisher Scientific), 50 μg/mL ascorbic acid-2-phosphate (Sigma), 4 × 10^-4^ M monothioglycerol (MTG, Nacalai), and 1× Insulin-Transferrin-Selenium solution (ITS-G, Thermo Fisher Scientific) (referred to as EB basal medium), 50 ng/mL recombinant human (rh) BMP-4 (R&D Systems), 50 ng/mL rhVEGF (R&D Systems), and 50 ng/mL bFGF (FujiFilm Wako) per well. After 24 h, 6 μM SB431542 (FujiFilm Wako) was added. After 4 days, the differentiating EBs were collected, washed, and resuspended in 2 mL EB basal medium supplemented with 50 ng/mL rhVEGF, 50 ng/mL rhbFGF and 50 ng/mL rhSCF (R&D Systems) per well and cultured for 2 days. After 7 days, the differentiating EBs were again collected, washed, and resuspended in 2 mL EB basal medium supplemented with 50 ng/mL rhVEGF, 50 ng/mL rhbFGF, 50 ng/mL rhSCF, 30 ng/mL rhTPO (PeproTech), and 10 ng/mL FLT3L (PeproTech) per well. From day 7, the cells were collected and replaced with fresh day 7 medium every 2–3 days. They were maintained in a 5% CO_2_/5% O_2_/90% N_2_ environment for the first 7 days and in a 5% CO_2_ environment thereafter. The differentiated iHPCs were filtered through 40-μm cell strainers and cryopreserved at −80°C with TC-protector (Bio-Rad antibodies).

### 2D feeder-free differentiation of iHPCs to T cell progenitors

We used previously reported iHPCs derived from TKT3v1-7 (13). Briefly, T-cell differentiation was induced on rhDL4-coated plates prepared one day prior to the iHPC seeding. rhDL4/Fc chimera protein solution (5 μg/mL, Sino Biological) was diluted with an equal volume of retronectin (5 μg/mL, TAKARA, Japan), 150 μL of the solution was added to each well of 48-well plates, and the plates were incubated overnight at 4°C. The coating solution was removed just before adding T-cell differentiation medium.

For iHPC seeding, 11-14 EBs were collected and dissociated into single cells by TryPLE Select (Thermo Fisher Scientific) treatment. A total of 2000 CD34+/CD43+ cells were FACS-sorted directly into the wells of a DL4-coated plate containing T-cell differentiation medium composed of αMEM (Thermo Fisher Scientific) supplemented with 15% FBS (Corning), 100× ITS-G (1×), 55 μM 2-Mercaptoethanol (Thermo Fisher Scientific), 50 μg/mL ascorbic acid-2-phosphate, 2 mM Glutamax, 50 ng/mL rhSCF, 50 ng/mL rhTPO, 50 ng/mL rhIL-7, 50 ng/mL FLT3L, 30 nM rhSDF-1α (PeproTech), and 15 μM SB203580 (Tocris Bioscience). A major portion of the medium (80%) was changed every other day. The differentiating cells were transferred to a new DL4-coated plate on day 7, and 1-2 × 10^5^ cells/well were transferred to a new DL4-coated plate on day 14. Cultures were maintained in a 5% CO_2_ environment.

### Maturation of T cell progenitors to CD8 single positive T cells

Day 21 DL4 cells were stimulated with a monoclonal antibody for CD3 (clone: OKT3, eBioscience) at 500 ng/mL in maturation medium composed of αMEM, 15% FBS, 100× ITS-G (1×), 50 μg/mL ascorbic acid-2-phosphate, 100× PSG (1×, Sigma), 10 ng/mL rhIL-7, 10 ng/mL rhIL-2 (Pepro-Tech), and 10 nM dexamethasone (Fuji Pharma). The cells were collected, washed, and resuspended in maturation medium without OKT3 after 3 days and incubated for 4 days in an environment containing 5% CO_2_ at 37°C.

### Maturation of iHPCs to CD4 and CD8 single positive T cells

We used a previously reported method with slight modifications (17). In brief, we made a dense MS5-hDLL4-iHPC mixture with 1 × 10^6^ MS5-hDLL4 cells and 1 × 10^5^ purified iHPCs in 1.5-mL microcentrifuge tubes. The mixture was adjusted to 15 μL per well. For each culture, a 0.4-μm Millicell Transwell insert (EMD Millipore, Billerica, MA; Cat. PICM0RG50) was placed in a 6-well plate containing 1.5 mL organoid culture medium per well. The medium was changed completely every 3-4 days.

### Cell staining with barcorded antibodies

Cells were stained with the following barcoded antibodies as previously described for CITE-seq (25): TotalSeq™-B0034 anti-human CD3 (UCHT1), TotalSeq™-B0072 anti-human CD4 (RPA-T4), and TotalSeq™-B0080 anti-human CD8a (RPA-T8). The antibodies were purchased from BioLegend (San Diego, CA, USA). Briefly, approximately 1.0 million cells per sample were resuspended in 1× CITE-seq staining buffer (PBS containing 1% BSA) and incubated for 10 min with Fc receptor block (TruStain FcX, BioLegend) to block FC receptor-mediated binding. Subsequently, the cells were incubated with mixtures of barcoded antibodies for 10 min at 4°C. The antibody concentrations were 0.5 μg for CD4 and 0.5 μg for CD8a per test, as recommended by the manufacturer (BioLegend) for PBMC applications. After staining, the cells were washed three times by resuspension in CITE-seq staining buffer, followed by centrifugation (400*g* for 5 min at 4°C) and supernatant exchange. After the final wash, the cells were resuspended in PBS containing 2% BSA and filtered through 40 μm cell strainers.

### Single-cell RNA-seq

Isolated single cell suspensions were subjected to droplet-based massively parallel scRNA-seq using a Chromium Single Cell 50 Reagent Kit as per the manufacturer’s instructions (10x Genomics). Briefly, cell suspensions were loaded at 700-1,200 cells/μL with the aim to capture 3,000 cells per well. The 10x Chromium Controller generated GEM droplets, where each cell was labeled with a specific barcode, and each transcript was labeled with a unique molecular identifier (UMI) during reverse transcription. The barcoded cDNA was isolated via a Dynabeads MyOne SILANE bead cleanup mixture. The cDNA was amplified by PCR and purified via SPRI bead cleanup.

For gene expression libraries, 50 ng of amplified cDNA was used for the library preparation, which consisted of fragmentation, end repair, A-tailing, adaptor ligation and sample index PCR as per the manufacturer’s instructions. The libraries were sequenced on a NovaSeq sequencer (Illumina).

### Single cell RNA-seq data processing

Sequenced reads of organoid culture samples were aligned to human GRCh38 genome references and quantified gene and antibody-derived tag counts as UMIs using Cell Ranger v3.1.0 software (10x Genomics). We imported the UMI count matrices into R v3.6.1 software Seurat v3.1.1 package, and further data processing was performed using Seurat package. Cells with a percentage of mitochondrial gene counts more than 20% were removed from further analysis as dead or damaged cells. Gene and antibody-derived tag counts were normalized by the global-scale normalization method and centered log-ratio normalization method, respectively. Variable genes within cells were selected by the “vst” method in Seurat package, and a principal component analysis was performed. The calculated principal components were used for the dimensional reduction and graph-based clustering. Then we performed a reference component analysis using RCA v1.0.0 or RCAv2 v2.0.0 package (44) to estimate tissue or cell types whose expression profiles were similar to that of each cell.

The datasets of the organoid culture were merged, and data processing including normalization, dimensional reduction, clustering, and reference component analysis, was performed. The objective cell populations were extracted according to the CITE-seq expression of CD4 and/or CD8A for the original data.

We downloaded BAM files of human thymocytes samples (GSE148978) from the SRA repository. BAM files were converted to FASTQ sequencing files using bamtofastq software v1.3.2 (10x Genomics). Sequenced reads were aligned to human GRCh38 genome reference, and gene tag counts were quantified as UMIs using Cell Ranger v5.0.1 software. Cells with UMI counts under 200 or over 10000 or a percentage of mitochondrial gene counts more than 5% were removed from further analysis. The datasets of human thymocytes were integrated by Seurat’s standard integration method, and data processing was performed. Clusters that consisted of cells that had the UMI counts or derived from only one donor were removed.

Sequenced reads of 2D culture samples were aligned to human GRCh38 genome references and quantified gene tag counts as UMIs using Cell Ranger software v5.0.1. Data processing was performed using Seurat v3.2.3. package.

The datasets of organoid culture, 2D culture and human thymocytes samples were integrated by the Seurat’s standard integration method, and data processing was performed.

Differentially expressed genes in a cluster relative to the other clusters were defined as having a fold change over 2 and p-value determined by Wilcoxon’s rank sum test less than 0.05.

### Pseudotime analysis

The integrated datasets were converted to monocle3 v0.2.3.0 package (45–48) format, and data processing was performed. Seurat’s embedding space of UMAP was used for further analysis. We acquired the trajectory graph by fitting a principal UMAP embedding space. The pseudotime was determined by setting the nodes of DN cells as the start. We extracted differentially expressed genes in the different paths through the trajectory using Moran’s I test, and the identified differentially expressed genes were classified in gene set modules of co-regulated genes along with the trajectory and pseudotime.

## Data availability statement

The data presented in the study are deposited in the SRA repository, accession number PRJNA1049373.

## Ethics statement

Ethical approval was not required for the study involving humans in accordance with the local legislation and institutional requirements. Written informed consent to participate in this study was not required from the participants or the participants’ legal guardians/next of kin in accordance with the national legislation and the institutional requirements. Ethical approval was not required for the studies on animals in accordance with the local legislation and institutional requirements because only commercially available established cell lines were used.

## Author contributions

YI: Writing – original draft, Conceptualization, Data curation, Formal Analysis, Investigation, Methodology. SI: Writing – original draft, Writing – review & editing, Conceptualization, Methodology, Supervision. ShA: Writing – original draft, Formal Analysis. TS: Writing – review & editing. SS: Writing – original draft. SuA: Writing – original draft. YK: Writing – review & editing, Conceptualization, Supervision. YS: Writing – review & editing. KO: Writing – review & editing. SK: Writing – review & editing, Writing – original draft, Conceptualization, Funding acquisition, Supervision.
