# Supplementary material for: Lineage tracing of T cell differentiation from T-iPSC by 2D feeder-free culture and 3D organoid culture
Source: Front Immunol. 2023 Dec 15;14:1303713. doi: 10.3389/fimmu.2023.1303713 (PMC10757342; doi:10.3389/fimmu.2023.1303713)
Supplement: Supplementary file 1 [file DataSheet_1.pdf]

Table S1	top_10_genes_3D_6w_Thymocyte_2D						
	p_val	avg_log2FC	pct.1	pct.2	p_val_adj	cluster	gene
1	0	1.13588664	0.266	0.056	0	0	CTSL
2	0	0.88916395	0.528	0.326	0	0	SELL
3	0	0.80300048	0.368	0.175	0	0	FCGRT
4	0	0.73875797	0.528	0.277	0	0	GIMAP4
5	1.28E-270	0.7502741	0.964	0.913	6.01E-266	0	RPL13A
6	1.30E-183	0.73417015	0.836	0.731	6.11E-179	0	RPL27A
7	2.33E-176	0.85843904	0.476	0.332	1.09E-171	0	RPS10
8	3.89E-175	0.8213431	0.304	0.167	1.82E-170	0	LGALS1
9	2.70E-86	0.69904655	0.728	0.639	1.26E-81	0	RPS17
10	4.79E-82	0.7915246	0.27	0.18	2.25E-77	0	KLRB1
11	0	1.7932873	0.557	0.096	0	1	LINC02446
12	0	1.26080957	0.312	0.045	0	1	PASK
13	0	1.18099507	0.861	0.523	0	1	IL7R
14	0	1.13995928	0.434	0.114	0	1	LINC00861
15	0	1.01178804	0.519	0.202	0	1	PCED1B-AS1
16	0	0.90135369	0.774	0.387	0	1	CCR7
17	0	0.88114684	0.455	0.176	0	1	LRRN3
18	0	0.76842374	0.738	0.471	0	1	GAS5
19	0	0.75738153	1	0.981	0	1	RPS18
20	0	0.67899064	1	0.996	0	1	RPLP1
21	0	2.73196244	0.973	0.682	0	2	ITM2A
22	0	1.83949085	0.933	0.541	0	2	SATB1
23	0	1.76518552	0.607	0.188	0	2	CCR9
24	0	1.51554262	0.498	0.139	0	2	CD40LG
25	0	1.43601853	0.556	0.277	0	2	TRAT1
26	0	1.43204317	0.707	0.286	0	2	TOX2
27	0	1.22491742	0.414	0.158	0	2	LZTFL1
28	0	1.15022221	0.706	0.405	0	2	MARCKSL1
29	0	1.1213914	0.545	0.247	0	2	BACH2
30	2.80E-277	1.24157124	0.264	0.105	1.31E-272	2	HPGD
31	0	1.08935562	0.799	0.443	0	3	CD8B
32	0	1.0532249	0.772	0.424	0	3	CD8A
33	2.38E-293	0.89297085	0.665	0.483	1.12E-288	3	GZMM
34	2.21E-276	1.02149357	0.37	0.152	1.04E-271	3	LINC02446

35	5.24E-215	0.68492959	0.738	0.602	2.46E-210	3	CHI3L2
36	5.57E-135	0.69185566	0.535	0.404	2.61E-130	3	ABLIM1
37	1.28E-124	0.69114898	0.494	0.373	6.02E-120	3	PAXX
38	9.18E-124	0.82447362	0.26	0.136	4.31E-119	3	STAG3
39	1.33E-98	0.68087073	0.259	0.149	6.25E-94	3	AOAH
40	1.28E-80	0.66891997	0.49	0.401	5.98E-76	3	CXCR4
41	0	1.92260349	0.746	0.255	0	4	S100A4
42	0	1.43689992	0.839	0.594	0	4	IL32
43	0	1.33493669	0.309	0.083	0	4	SESN1
44	0	1.30191692	0.397	0.12	0	4	BIRC3
45	1.12E-278	1.87221632	0.479	0.223	5.27E-274	4	NEAT1
46	2.35E-159	1.28374853	0.381	0.184	1.10E-154	4	KLRB1
47	4.31E-151	1.23600139	0.477	0.279	2.02E-146	4	NFKBIA
48	1.46E-76	1.27573573	0.269	0.151	6.84E-72	4	JUND
49	2.81E-60	1.12736975	0.378	0.27	1.32E-55	4	ARL4C
50	6.39E-56	1.18030815	0.262	0.159	3.00E-51	4	LST1
51	0	3.67165186	0.604	0.064	0	5	CCL5
52	0	3.05939813	0.776	0.141	0	5	NKG7
53	0	2.6659469	0.293	0.015	0	5	CCL4
54	0	2.61618602	0.39	0.022	0	5	GZMK
55	0	2.5946998	0.638	0.101	0	5	GZMA
56	0	2.54861376	0.712	0.082	0	5	CTSW
57	0	2.19654881	0.647	0.064	0	5	CST7
58	0	2.01941486	0.445	0.04	0	5	TYROBP
59	0	1.94825676	0.609	0.079	0	5	XCL2
60	0	1.93789103	0.577	0.079	0	5	XCL1
61	0	4.00521615	0.95	0.12	0	6	CD1E
62	0	3.42137138	0.837	0.108	0	6	MZB1
63	0	3.37055114	0.666	0.047	0	6	RAG1
64	0	3.22605894	0.796	0.101	0	6	CD1B
65	0	3.16530493	0.724	0.053	0	6	ARPP21
66	0	2.98591677	0.707	0.056	0	6	DNTT
67	0	2.94742665	0.678	0.082	0	6	CD1A
68	0	2.91970502	0.675	0.056	0	6	ELOVL4
69	0	2.90335425	0.613	0.051	0	6	CD1C
70	0	2.79149533	0.584	0.031	0	6	RAG2

71	0	1.72730437	0.899	0.42	0	7	CD74
72	0	1.58686176	0.355	0.014	0	7	NDNF
73	0	1.52615394	0.607	0.21	0	7	SMC4
74	0	1.22050094	0.389	0.101	0	7	SMIM24
75	0	1.15124414	0.805	0.438	0	7	CD27
76	0	1.03107485	0.25	0.036	0	7	GNG8
77	1.06E-293	1.20180726	0.608	0.283	4.98E-289	7	DNPH1
78	3.58E-263	1.03500565	0.367	0.114	1.68E-258	7	TNFRSF1B
79	2.56E-260	1.06678648	0.788	0.433	1.20E-255	7	ARMH1
80	2.90E-197	1.05262645	0.517	0.251	1.36E-192	7	M6PR
81	0	3.063825	0.496	0.032	0	8	NR4A1
82	0	2.13788513	0.329	0.041	0	8	NFKBID
83	0	2.0897044	0.699	0.226	0	8	DUSP2
84	0	1.53184784	0.39	0.077	0	8	EGR1
85	3.85E-304	1.692864	0.477	0.161	1.81E-299	8	REL
86	1.46E-255	1.21459444	0.649	0.302	6.84E-251	8	SDCBP
87	1.16E-226	1.32268964	0.712	0.407	5.42E-222	8	SRGN
88	1.19E-219	1.22339196	0.676	0.353	5.58E-215	8	CD69
89	2.00E-120	1.30260713	0.601	0.39	9.38E-116	8	PRDX1
90	3.22E-69	1.33748121	0.267	0.133	1.51E-64	8	BIRC3
91	0	2.24907081	0.63	0.23	0	9	DUSP2
92	0	2.15509486	0.652	0.262	0	9	ITGA4
93	0	2.09453785	0.515	0.1	0	9	CD1A
94	0	1.93566838	0.599	0.214	0	9	MIR181A1HG
95	0	1.79176721	0.616	0.233	0	9	SLAMF1
96	0	1.77339965	0.7	0.311	0	9	SH2D1A
97	0	1.6466744	0.884	0.669	0	9	LEF1
98	0	1.54731623	0.39	0.07	0	9	CD1C
99	3.98E-297	1.55536721	0.503	0.179	1.87E-292	9	LZTFL1
100	8.38E-293	1.49624134	0.583	0.23	3.93E-288	9	CCR9
101	0	2.41970238	0.745	0.177	0	10	LGALS1
102	0	2.27275926	0.742	0.264	0	10	HMGB2
103	0	2.17146513	0.521	0.081	0	10	ATP5E
104	0	2.15912404	0.482	0.026	0	10	KIAA0101
105	0	1.90546087	0.521	0.081	0	10	GNB2L1
106	0	1.90328081	0.447	0.028	0	10	TOP2A

107	0	1.80764848	0.52	0.081	0	10	ATP5L
108	0	1.8010202	0.825	0.346	0	10	RPS10
109	1.57E-307	2.14051905	0.908	0.457	7.37E-303	10	TUBA1B
110	3.00E-85	2.13205601	0.881	0.65	1.41E-80	10	HIST1H4C
111	0	3.88717181	0.642	0.032	0	11	TRDV1
112	0	3.8866457	0.811	0.06	0	11	TRDC
113	0	2.01361019	0.685	0.044	0	11	RTKN2
114	0	1.86936286	0.316	0.008	0	11	TRGV4
115	0	1.64949541	0.648	0.144	0	11	TRG-AS1
116	0	1.59416256	0.306	0.015	0	11	MYOM2
117	0	1.44014439	0.623	0.103	0	11	SMIM24
118	4.10E-299	1.49794185	0.651	0.182	1.92E-294	11	IKZF2
119	4.91E-260	1.57016461	0.756	0.268	2.30E-255	11	ITGA4
120	5.92E-190	1.53014788	0.289	0.054	2.78E-185	11	TRGV3
121	0	4.49868892	0.996	0.057	0	12	PTCRA
122	0	3.66710904	0.959	0.04	0	12	FXYD2
123	0	3.2618737	0.991	0.312	0	12	MAL
124	0	3.09473898	1	0.614	0	12	CD99
125	0	3.06312204	0.996	0.135	0	12	MZB1
126	0	2.96836719	0.978	0.078	0	12	DNTT
127	0	2.65779973	0.858	0.044	0	12	AC011893.1
128	0	2.52924013	0.941	0.077	0	12	ARPP21
129	0	2.4716794	0.953	0.132	0	12	GIHCG
130	0	2.43760127	0.604	0.011	0	12	JCHAIN
131	0	2.4909373	0.925	0.357	0	13	ISG15
132	0	2.29264481	0.819	0.16	0	13	MX1
133	0	2.19560236	0.586	0.031	0	13	IFIT1
134	0	1.90754794	0.869	0.296	0	13	LY6E
135	0	1.8582609	0.593	0.071	0	13	OAS1
136	0	1.76515653	0.449	0.055	0	13	RSAD2
137	0	1.7488381	0.67	0.135	0	13	IFI44L
138	4.30E-307	2.28436881	0.816	0.274	2.02E-302	13	IFI6
139	1.77E-231	1.78771071	0.836	0.358	8.31E-227	13	STAT1
140	1.41E-124	1.66921488	0.512	0.189	6.64E-120	13	MT2A
141	0	3.57834614	0.629	0.035	0	14	TNFRSF4
142	0	3.38805577	0.806	0.016	0	14	TIGIT

143	0	2.97439738	0.731	0.025	0	14	FOXP3
144	0	2.96200396	0.583	0.028	0	14	TNFRSF18
145	0	2.95033674	0.754	0.036	0	14	GBP5
146	0	2.49466068	0.56	0.002	0	14	LINC01943
147	2.61E-292	2.51215979	0.566	0.038	1.23E-287	14	CXCR6
148	1.47E-196	2.86892311	0.674	0.084	6.91E-192	14	BATF
149	1.37E-162	2.54757081	0.806	0.151	6.44E-158	14	CARD16
150	1.08E-98	2.88758649	0.989	0.609	5.08E-94	14	IL32
