# Supplementary material for: Lineage tracing of T cell differentiation from T-iPSC by 2D feeder-free culture and 3D organoid culture
Source: Front Immunol. 2023 Dec 15;14:1303713. doi: 10.3389/fimmu.2023.1303713 (PMC10757342; doi:10.3389/fimmu.2023.1303713)
Supplement: Supplementary file 2 [file DataSheet_2.pdf]

Supplemental Figure 1. Characters of iHPCs and 2D-culture immature T-iPS-T cells.
(A) CD34+, CD43+ population were extracted as iHPCs from feeder free EB formation.
(B) Flow cytometry plots of T-iPS-T differentiated on 2D culture for 21 days based on CD4 and CD8a expression.

Supplemental Figure 2. The correlation between CD8a and CD8b of T-iPS-T cells.
(A) T-iPS-T cells cultured in 2D.
(B) T-iPS-T cells cultured in 3D. This data emanated from an alternate 6 weeks sample.

Supplemental Figure 3. The archival flow cytometry datasets of the 3D organoid culture
Two datasets were presented for each week. The individual plots were delimited by viable cells and CD3+/abTCR+ expression.

Supplemental Figure 4. Flow cytometry plot of 1 week sample of 3D organoid culture
The plot depicting CD3+acTCR+ is presented in the left panel. The middle panel illustrates the expression of CD8a and CD4. The right panel displays the outcome of the unstained sample.

Supplemental Figure 5. scRNA-seq analysis of 3D culture
(A) UMAP plot of T-iPS-T cells in organoid culture at different weeks.
(B) RCA score of organoid culture at different weeks.
(C) RCA score of whole data organoid culture.
(D) Validation of the expression of T cell-related transcription marker genes in T cell lineage-committing cells from organoid culture. UMAP plots showing the representative expression of T cell-related transcription marker genes BCL11B, ZBTB7B, RUNX3, GATA3, RORC RAG1 and RAG2.

Supplemental Figure 6. scRNA-seq analysis of 2D and 3D combined datasets
(A) UMAP plot of T-iPS-T cells in 2D culture.
(B) Combined UMAP plot of T-iPS-T cells from 2D and 3D cultures.
(C) Feature plots highlighting the expression of the selected marker genes CD4, CD8a and CD8b.
(D) Feature plots of GZMK, CD69 and ITGAL in 2D and organoid cultures.

Supplemental Figure 7. Feature plots of 3D, thymocytes and 2D cultures
(A) Merged feature plots of 2D-cultured cells, 3D-cultured cells and thymocytes showing CD8A, CD8B, BCL11B, ZBTB7B, GATA3, RUNX3, and CD40LG expression.
(B) Feature plots of designated gene sets from 2D-cultured cells, 3D-cultured cells and thymocytes.

Supplemental Table 1. Top 10 genes of 6weeks cultured T-iPS-T in 3D organoid, human thymocyte dataset and T-iPS-T in 2D culture

Figure S1

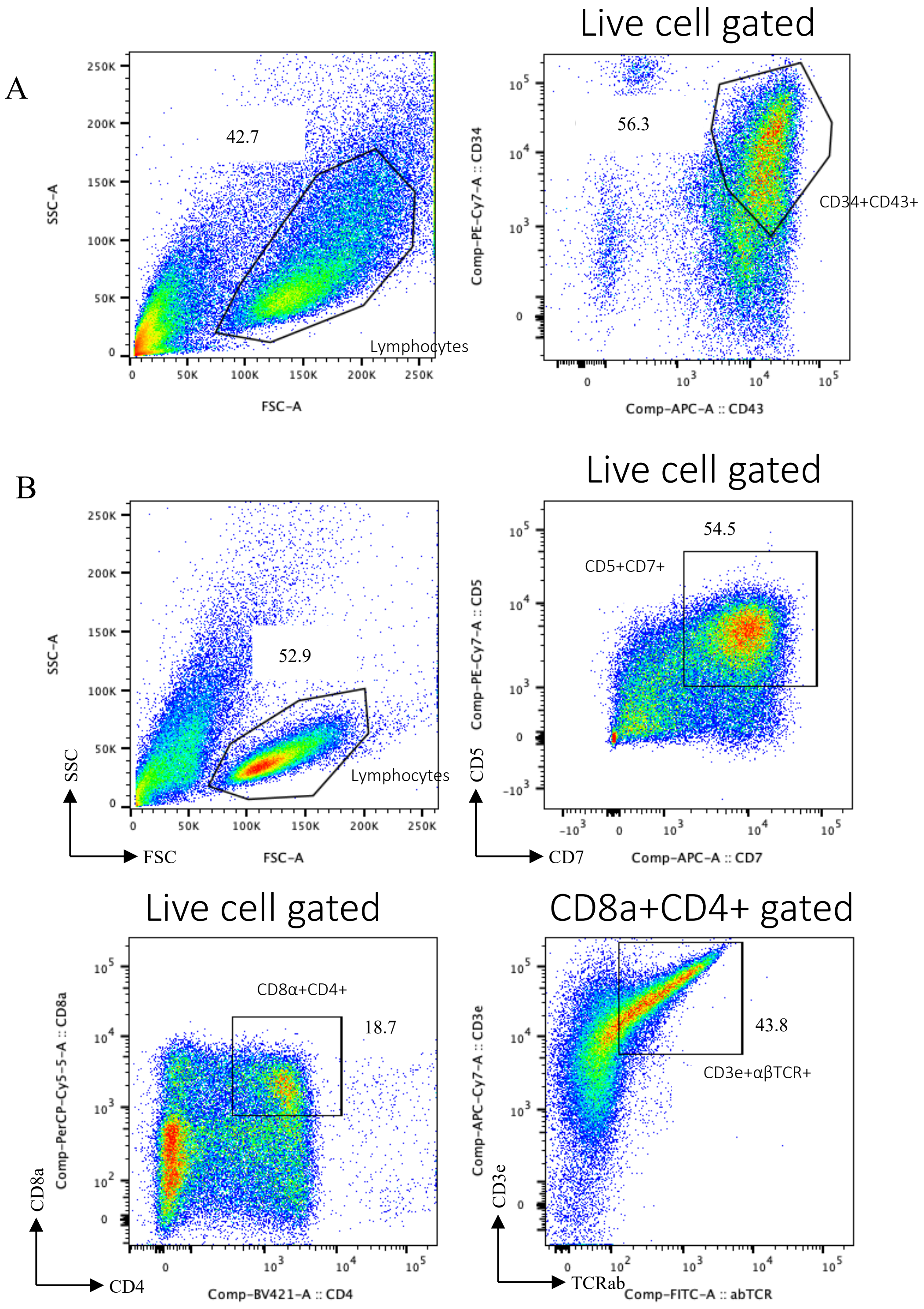

A
Live cell gated
42.7
Lymphocytes
SSC-A
FSC-A
56.3
CD34+CD43+
Comp-PE-Cy7-A :: CD34
Comp-APC-A :: CD43
B
Live cell gated
52.9
Lymphocytes
SSC
FSC
SSC-A
FSC-A
54.5
CD5+CD7+
CD5
CD7
Comp-PE-Cy7-A :: CD5
Comp-APC-A :: CD7
Live cell gated
CD8α+CD4+
18.7
CD8a
CD4
Comp-PerCP-Cy5-5-A :: CD8a
Comp-BV421-A :: CD4
CD8a+CD4+ gated
43.8
CD3e+αβTCR+
CD3e
TCRab
Comp-APC-Cy7-A :: CD3e
Comp-FITC-A :: abTCR

Figure S2

A

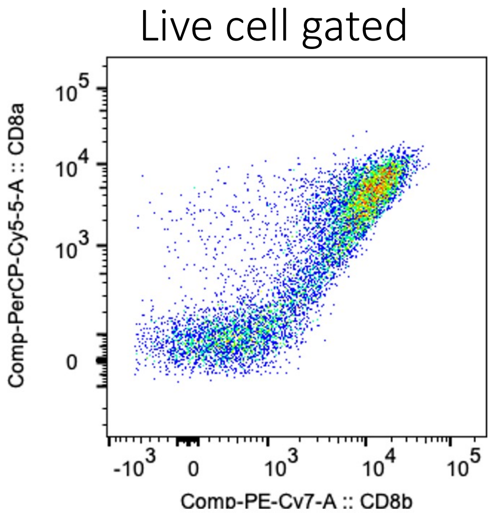

Live cell gated
Comp-PerCP-Cy5-5-A :: CD8a
Comp-PE-Cy7-A :: CD8b

B

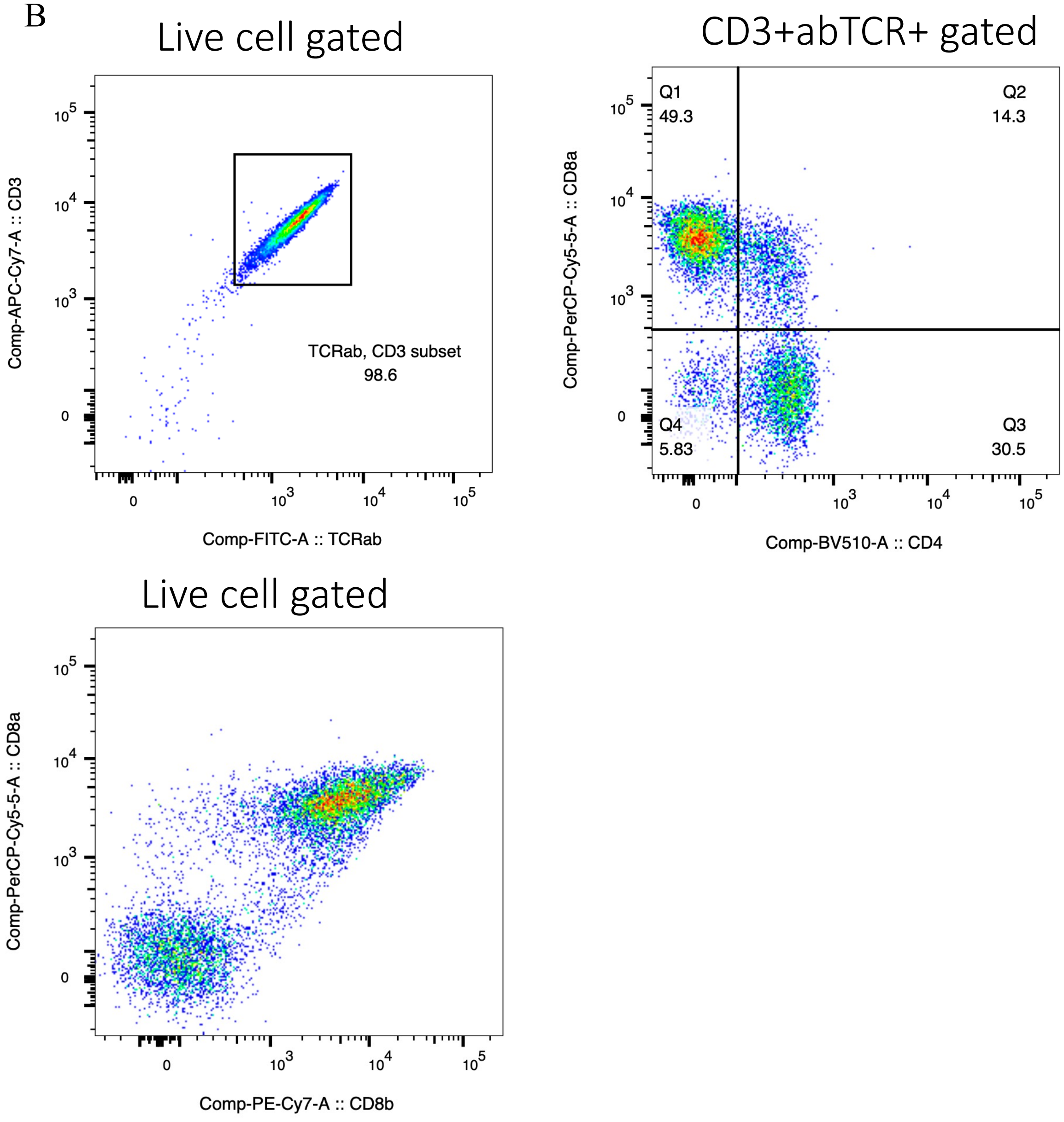

Live cell gated
Comp-APC-Cy7-A :: CD3
Comp-FITC-A :: TCRab
TCRab, CD3 subset
98.6
CD3+abTCR+ gated
Q1
49.3
Q2
14.3
Q3
30.5
Q4
5.83
Comp-PerCP-Cy5-5-A :: CD8a
Comp-BV510-A :: CD4
Live cell gated
Comp-PerCP-Cy5-5-A :: CD8a
Comp-PE-Cy7-A :: CD8b

Figure S3

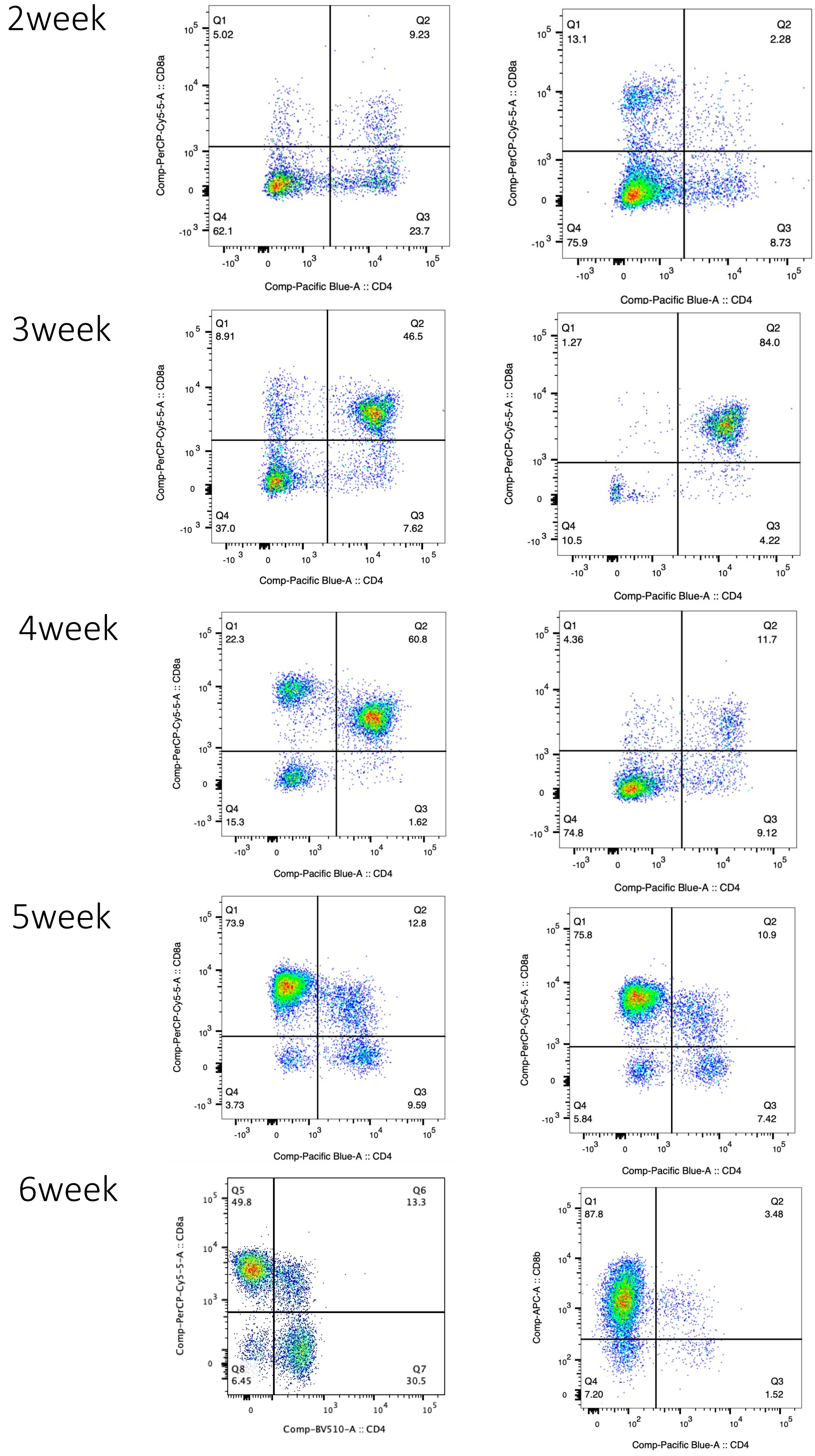
2week
Q1 5.02
Q2 9.23
Q4 62.1
Q3 23.7
Comp-PerCP-Cy5-5-A :: CD8a
Comp-Pacific Blue-A :: CD4
Q1 13.1
Q2 2.28
Q4 75.9
Q3 8.73
Comp-PerCP-Cy5-5-A :: CD8a
Comp-Pacific Blue-A :: CD4
3week
Q1 8.91
Q2 46.5
Q4 37.0
Q3 7.62
Comp-PerCP-Cy5-5-A :: CD8a
Comp-Pacific Blue-A :: CD4
Q1 1.27
Q2 84.0
Q4 10.5
Q3 4.22
Comp-PerCP-Cy5-5-A :: CD8a
Comp-Pacific Blue-A :: CD4
4week
Q1 22.3
Q2 60.8
Q4 15.3
Q3 1.62
Comp-PerCP-Cy5-5-A :: CD8a
Comp-Pacific Blue-A :: CD4
Q1 4.36
Q2 11.7
Q4 74.8
Q3 9.12
Comp-PerCP-Cy5-5-A :: CD8a
Comp-Pacific Blue-A :: CD4
5week
Q1 73.9
Q2 12.8
Q4 3.73
Q3 9.59
Comp-PerCP-Cy5-5-A :: CD8a
Comp-Pacific Blue-A :: CD4
Q1 75.8
Q2 10.9
Q4 5.84
Q3 7.42
Comp-PerCP-Cy5-5-A :: CD8a
Comp-Pacific Blue-A :: CD4
6week
Q5 49.8
Q6 13.3
Q8 6.45
Q7 30.5
Comp-PerCP-Cy5-5-A :: CD8a
Comp-BV510-A :: CD4
Q1 87.8
Q2 3.48
Q4 7.20
Q3 1.52
Comp-APC-A :: CD8b
Comp-Pacific Blue-A :: CD4

Figure S4

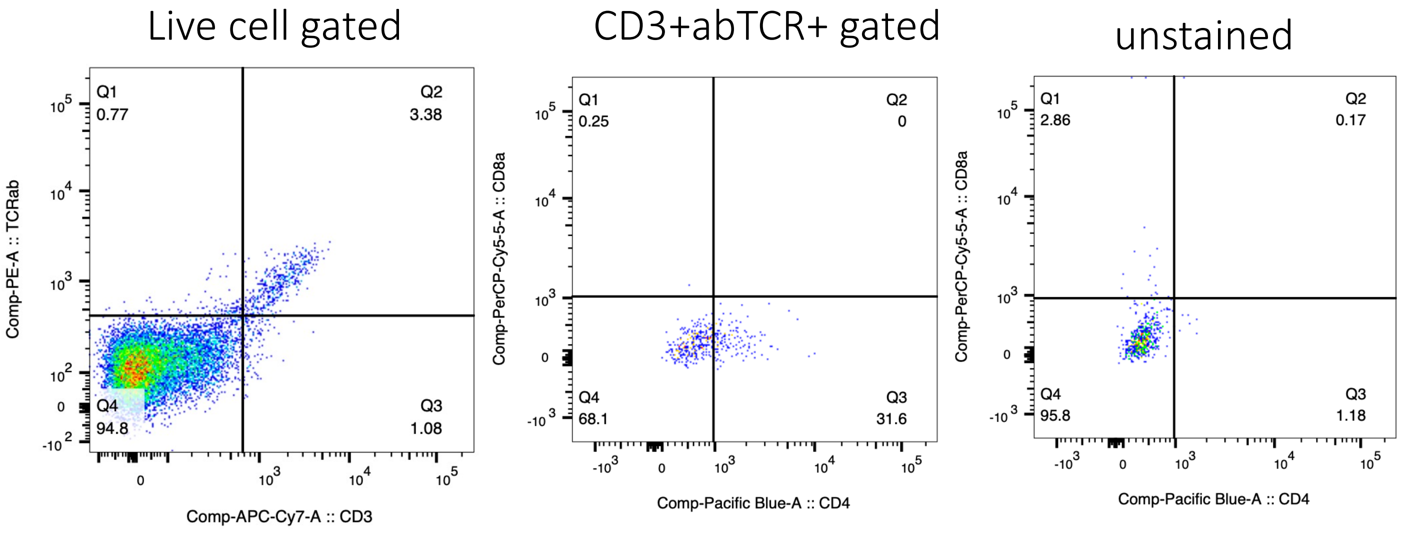
Live cell gated
CD3+abTCR+ gated
unstained
Q1
0.77
Q2
3.38
Q4
94.8
Q3
1.08
Comp-PE-A :: TCRab
Comp-APC-Cy7-A :: CD3
Q1
0.25
Q2
0
Q4
68.1
Q3
31.6
Comp-PerCP-Cy5-5-A :: CD8a
Comp-Pacific Blue-A :: CD4
Q1
2.86
Q2
0.17
Q4
95.8
Q3
1.18
Comp-PerCP-Cy5-5-A :: CD8a
Comp-Pacific Blue-A :: CD4

Figure S5

A

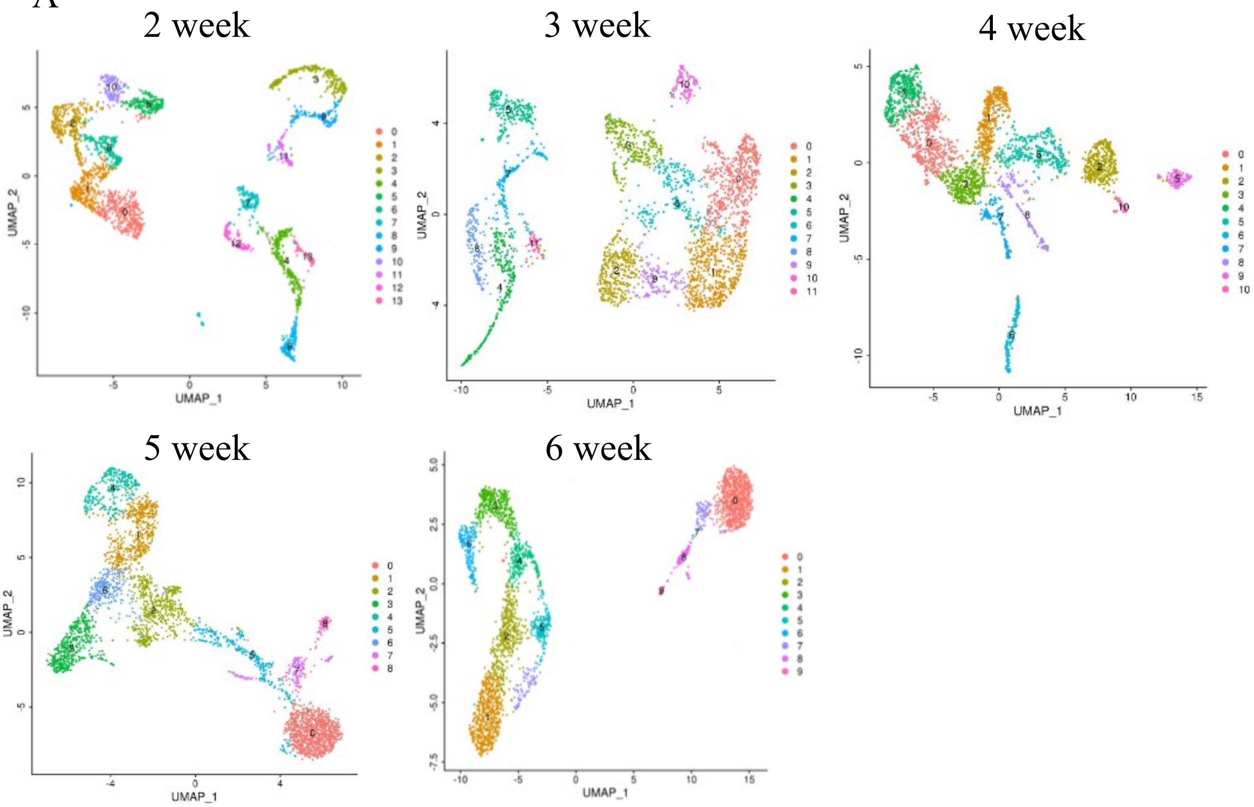

B

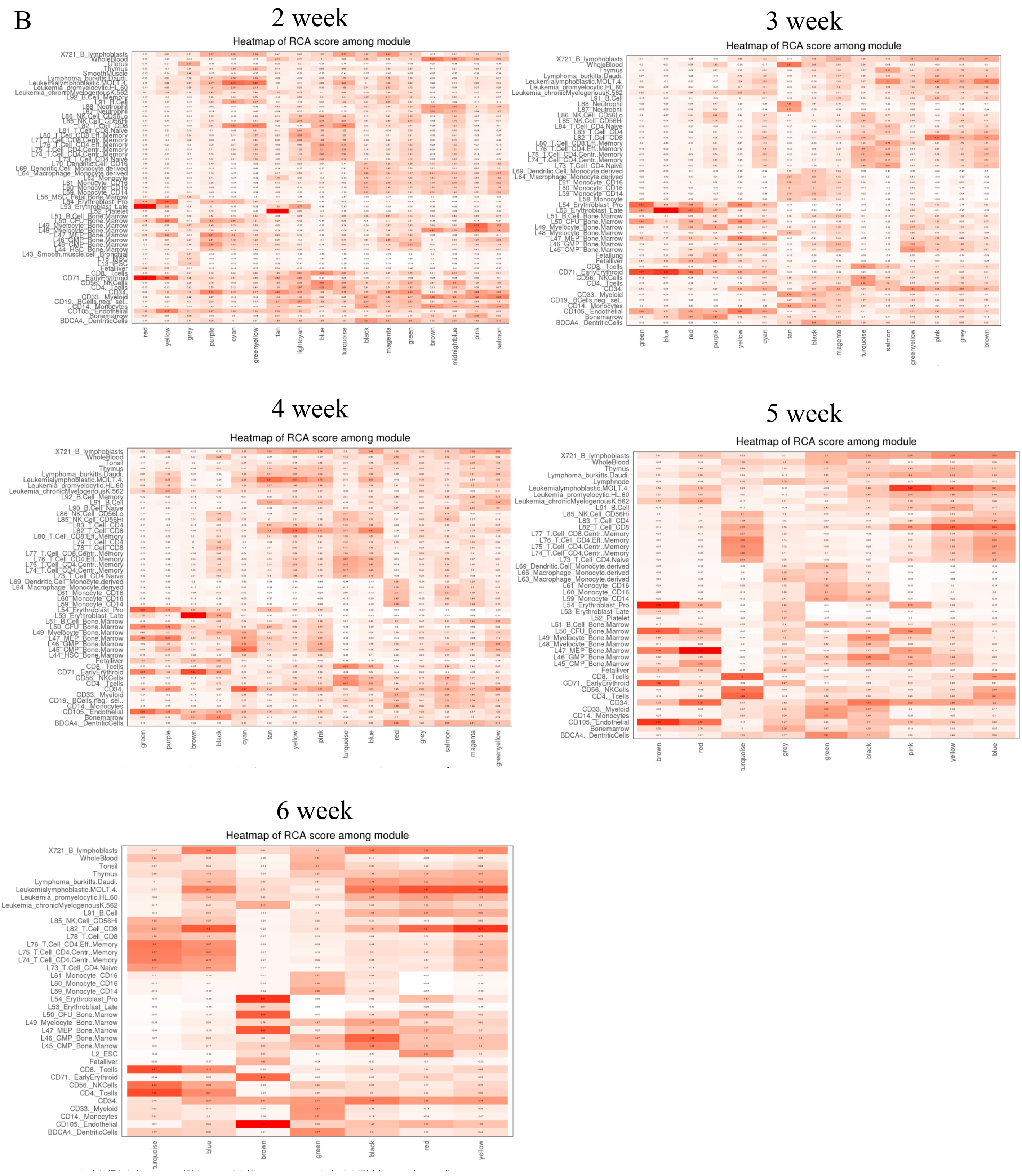
2 week
Heatmap of RCA score among module
red
yellow
grey
purple
cyan
greenyellow
tan
lightcyan
blue
turquoise
black
magenta
green
brown
midnightblue
pink
salmon
3 week
Heatmap of RCA score among module
green
blue
red
purple
yellow
cyan
tan
black
magenta
turquoise
salmon
greenyellow
pink
grey
brown
4 week
Heatmap of RCA score among module
green
purple
brown
black
cyan
tan
yellow
pink
turquoise
blue
red
grey
salmon
magenta
greenyellow
5 week
Heatmap of RCA score among module
brown
red
turquoise
grey
green
black
pink
yellow
blue
6 week
Heatmap of RCA score among module
X721_B_lymphoblasts
WholeBlood
Tonsil
Thymus
Lymphoma_burkitts.Daudi.
Leukemialymphoblastic.MOLT.4.
Leukemia_promyelocytic.HL.60
Leukemia_chronicMyelogenousK.562
L91_B.Cell
L85_NK.Cell_CD56Hi
L82_T.Cell_CD8
L78_T.Cell_CD8
L76_T.Cell_CD4.Eff..Memory
L75_T.Cell_CD4.Centr..Memory
L74_T.Cell_CD4.Centr..Memory
L73_T.Cell_CD4.Naive
L61_Monocyte_CD16
L60_Monocyte_CD16
L59_Monocyte_CD14
L54_Erythroblast_Pro
L53_Erythroblast_Late
L50_CFU_Bone.Marrow
L49_Myelocyte_Bone.Marrow
L47_MEP_Bone.Marrow
L46_GMP_Bone.Marrow
L45_CMP_Bone.Marrow
L2_ESC
Fetalliver
CD8._Tcells
CD71._EarlyErythroid
CD56._NKCells
CD4._Tcells
CD34.
CD33._Myeloid
CD14._Monocytes
CD105._Endothelial
BDCA4._DentriticCells
turquoise
blue
brown
green
black
red
yellow

C

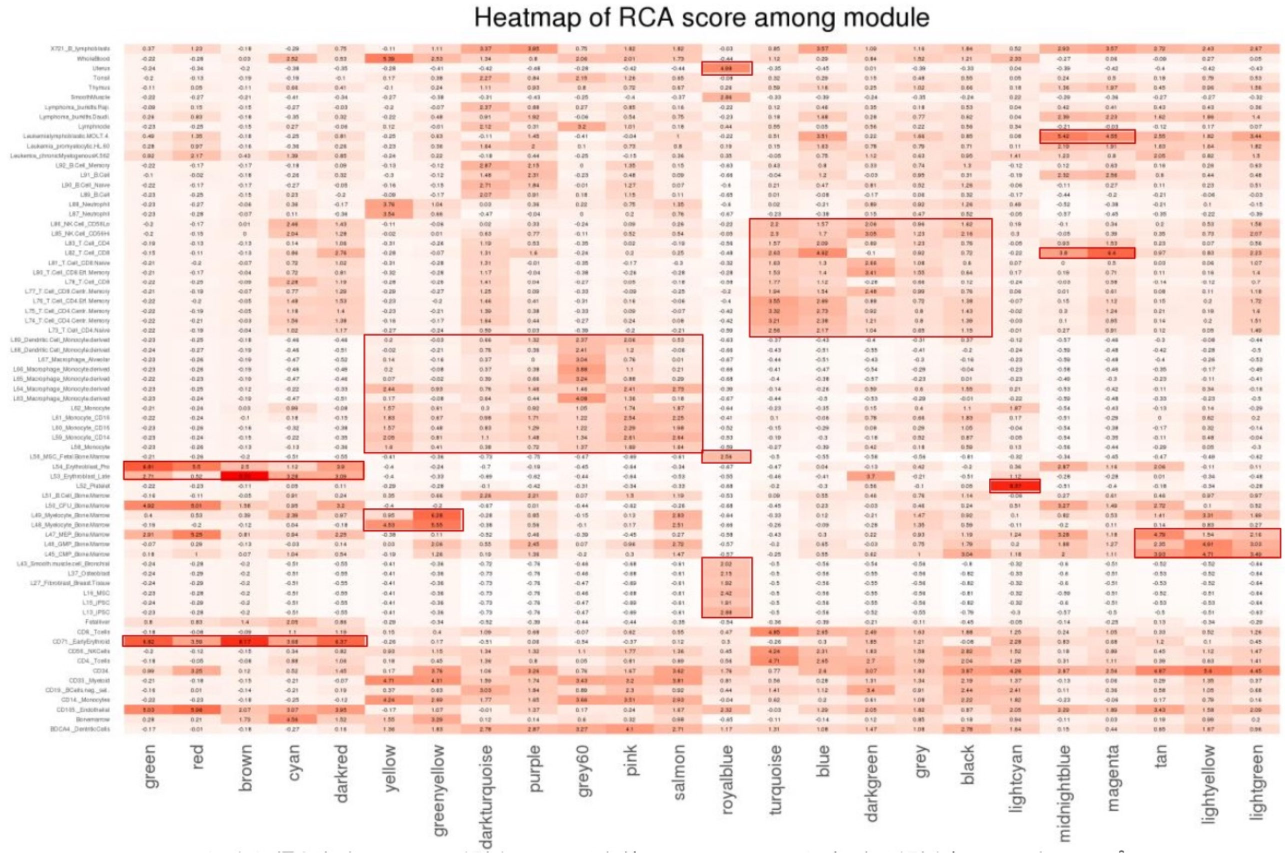
Heatmap of RCA score among module
green
red
brown
cyan
darkred
yellow
greenyellow
darkturquoise
purple
grey60
pink
salmon
royalblue
turquoise
blue
darkgreen
grey
black
lightcyan
midnightblue
magenta
tan
lightyellow
lightgreen

D

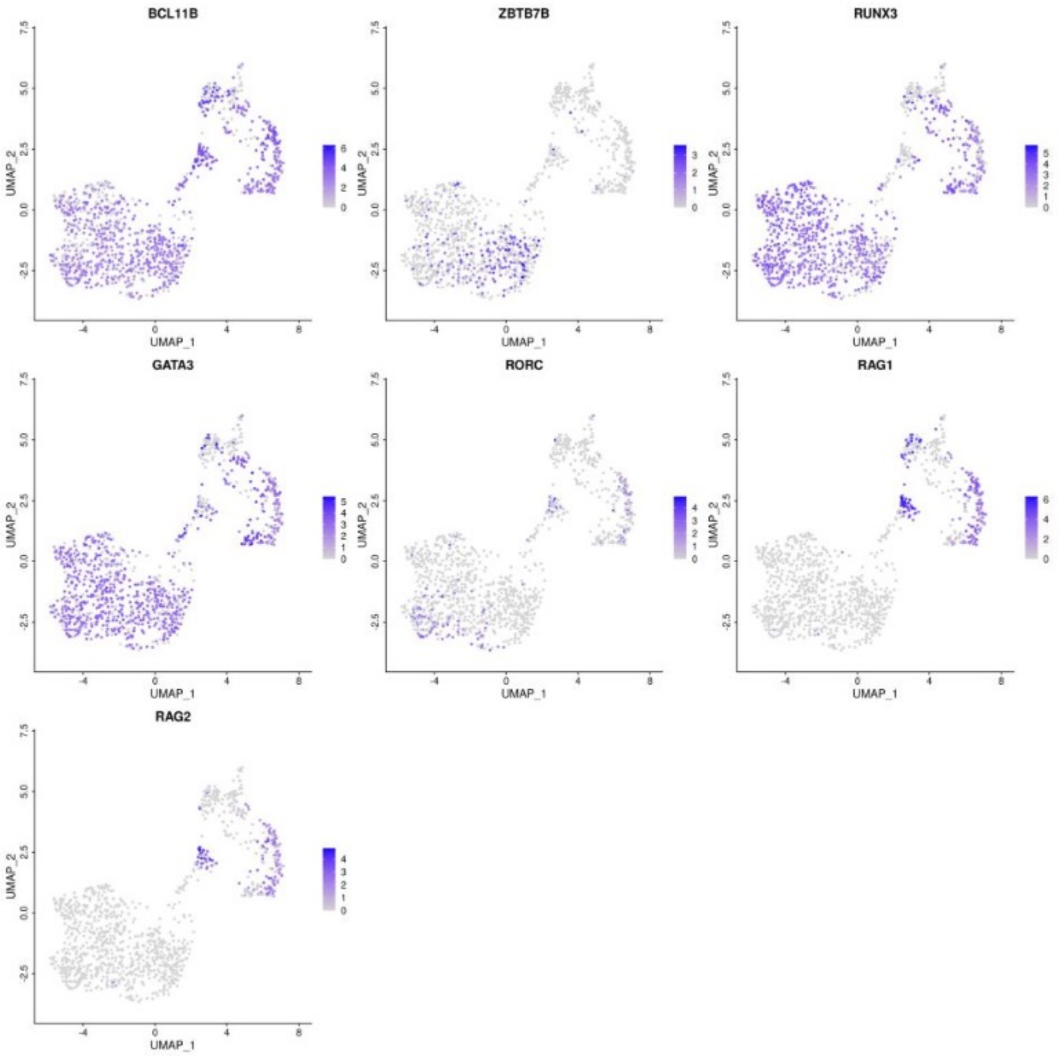

Figure S6

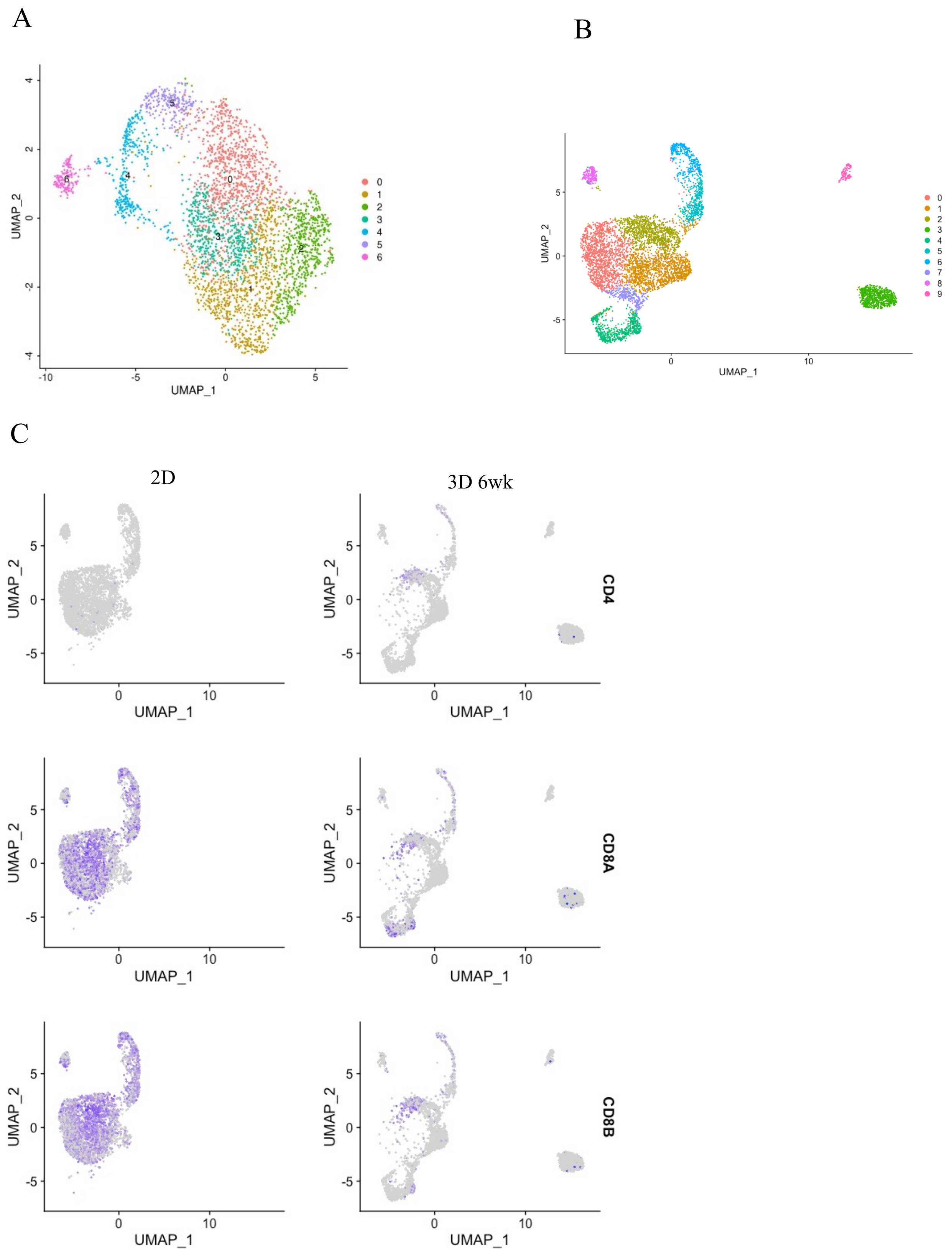

D

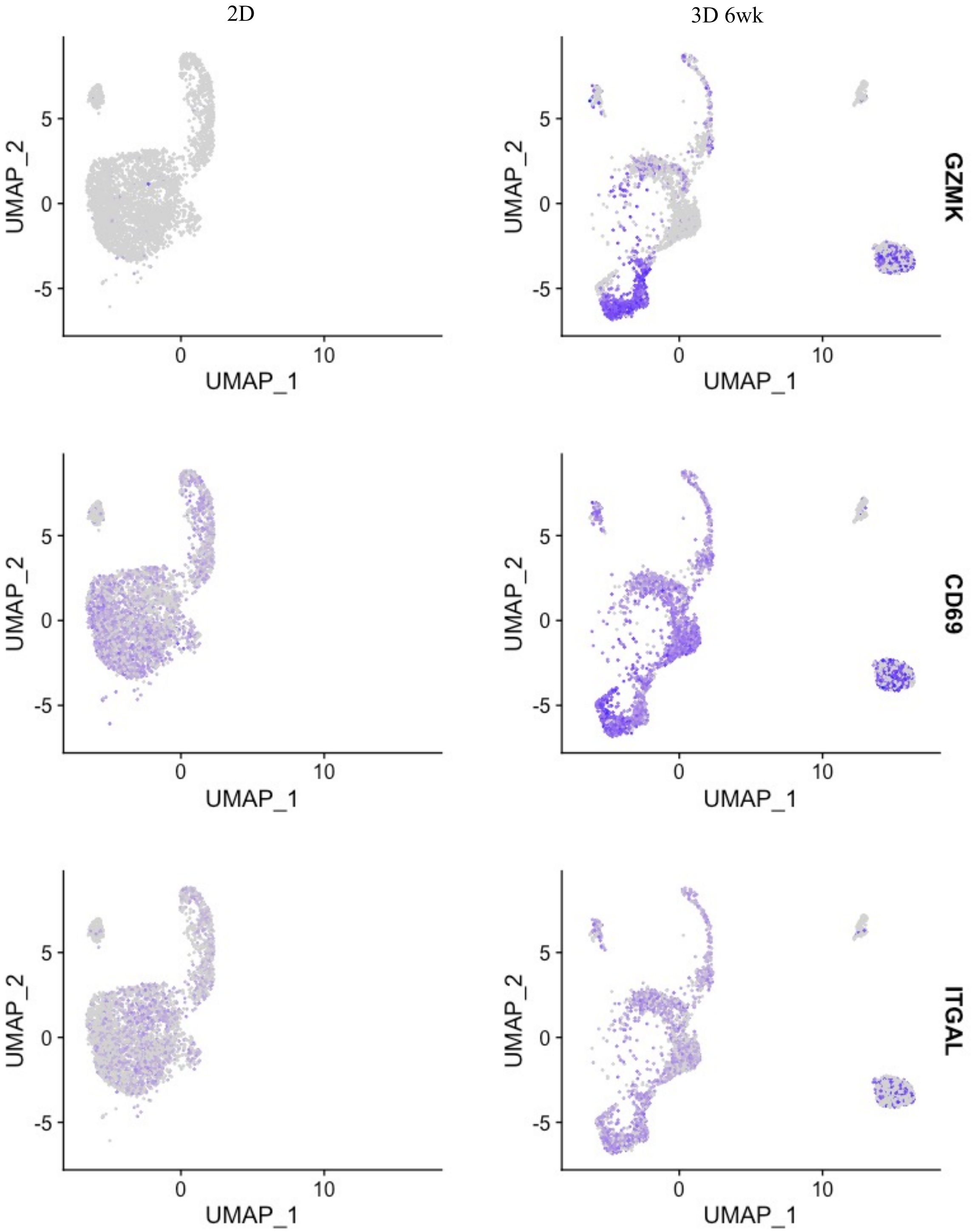

Figure S7 A

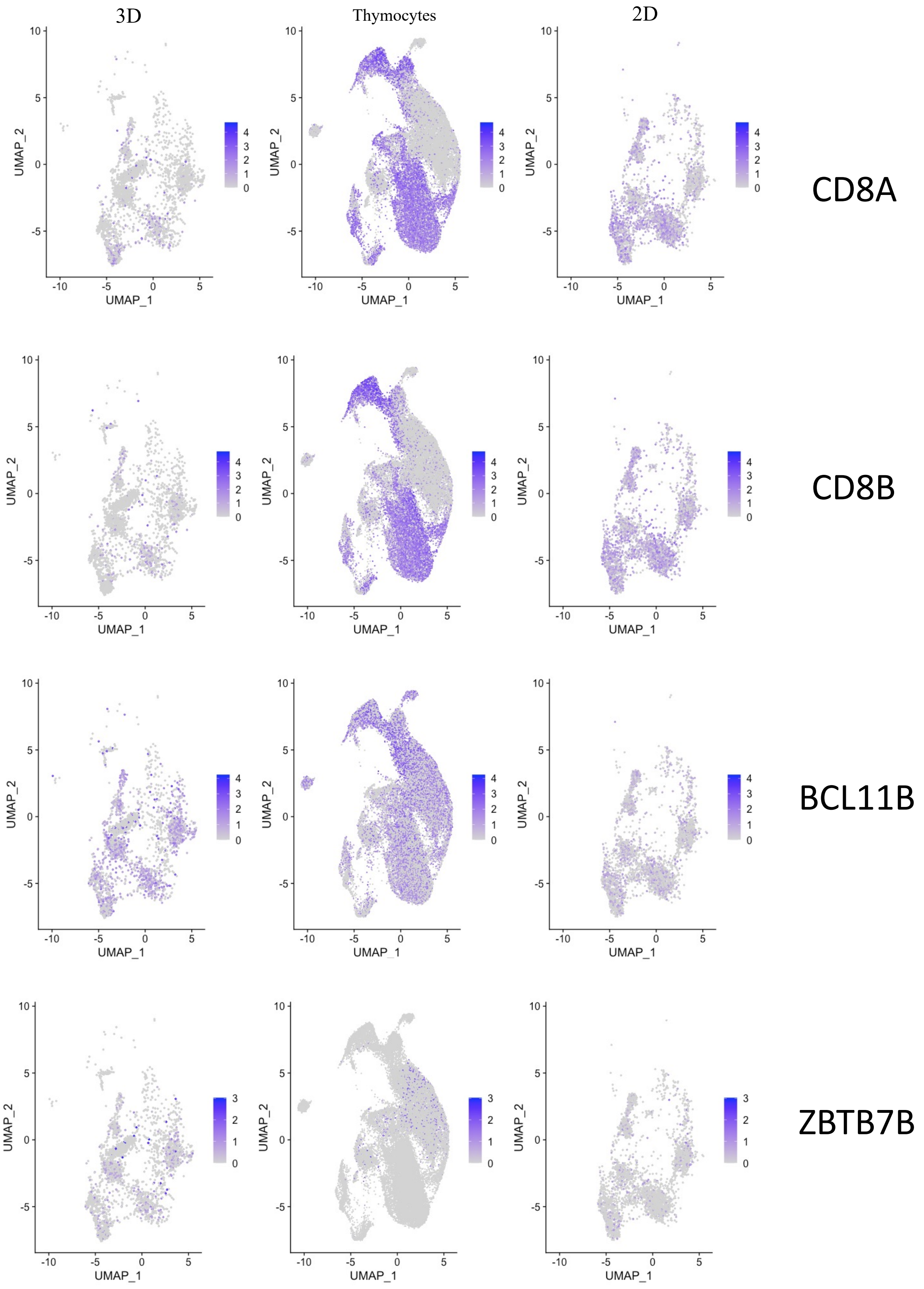

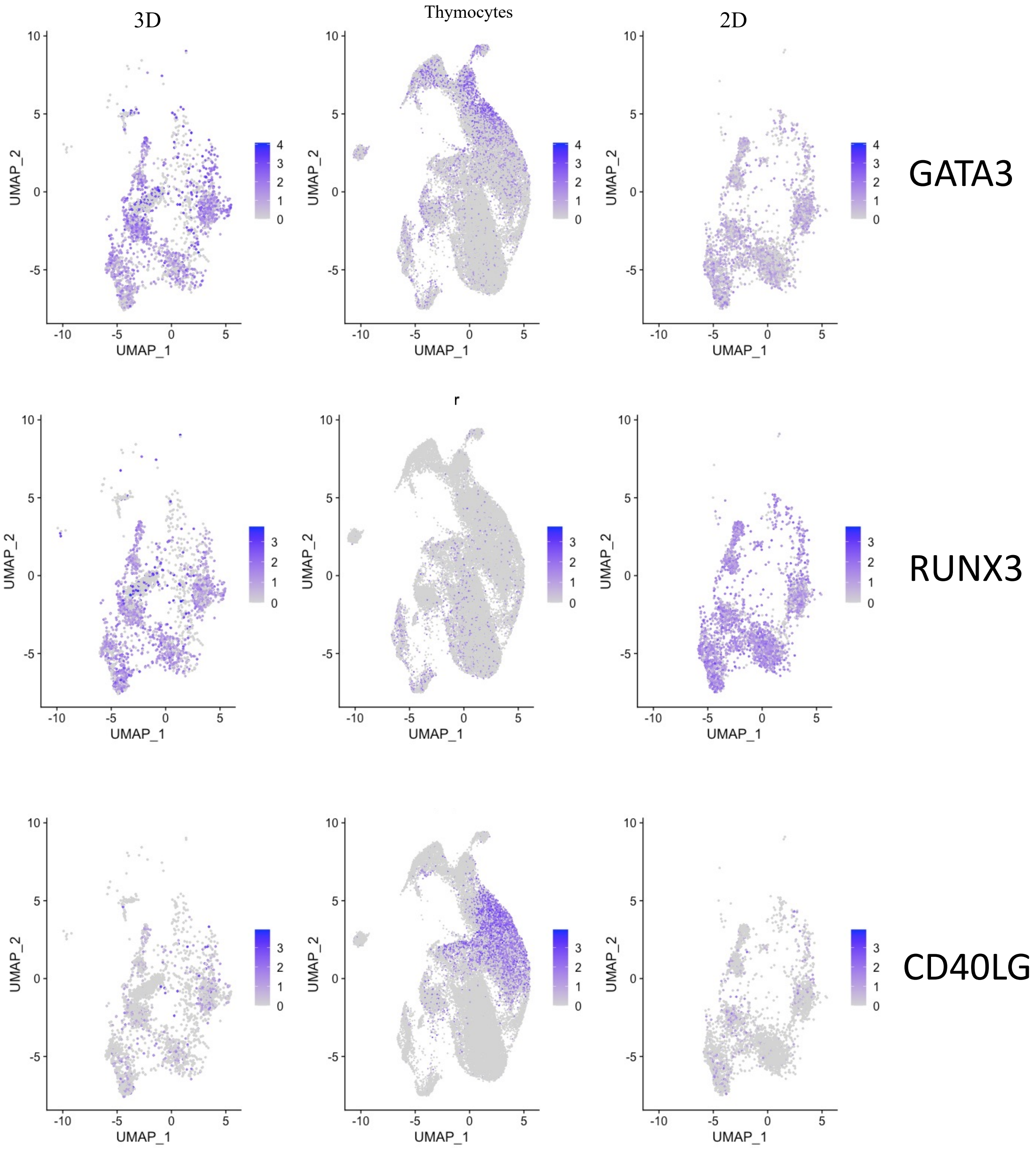
3D
Thymocytes
2D
GATA3
r
RUNX3
CD40LG
UMAP_1
UMAP_2

B

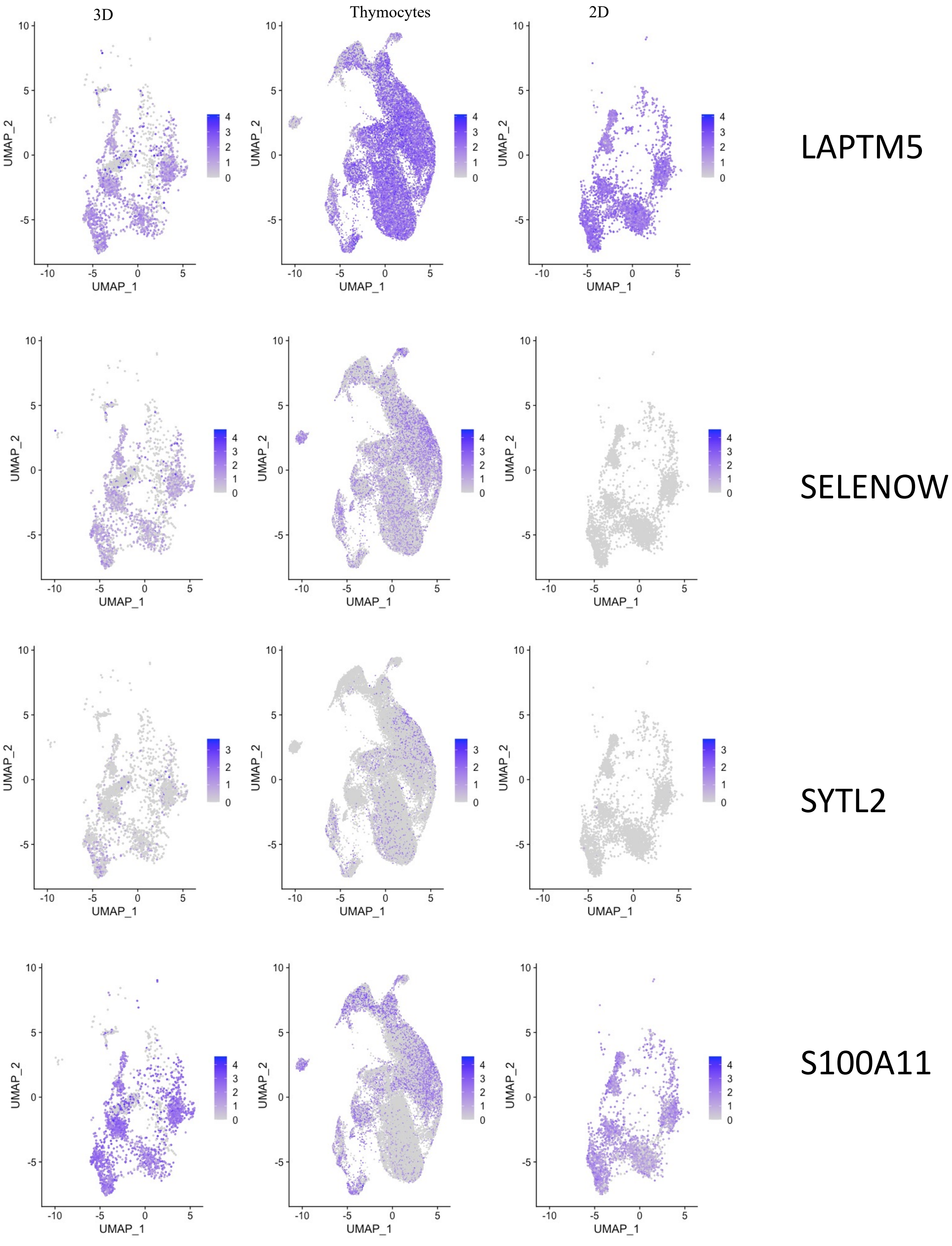

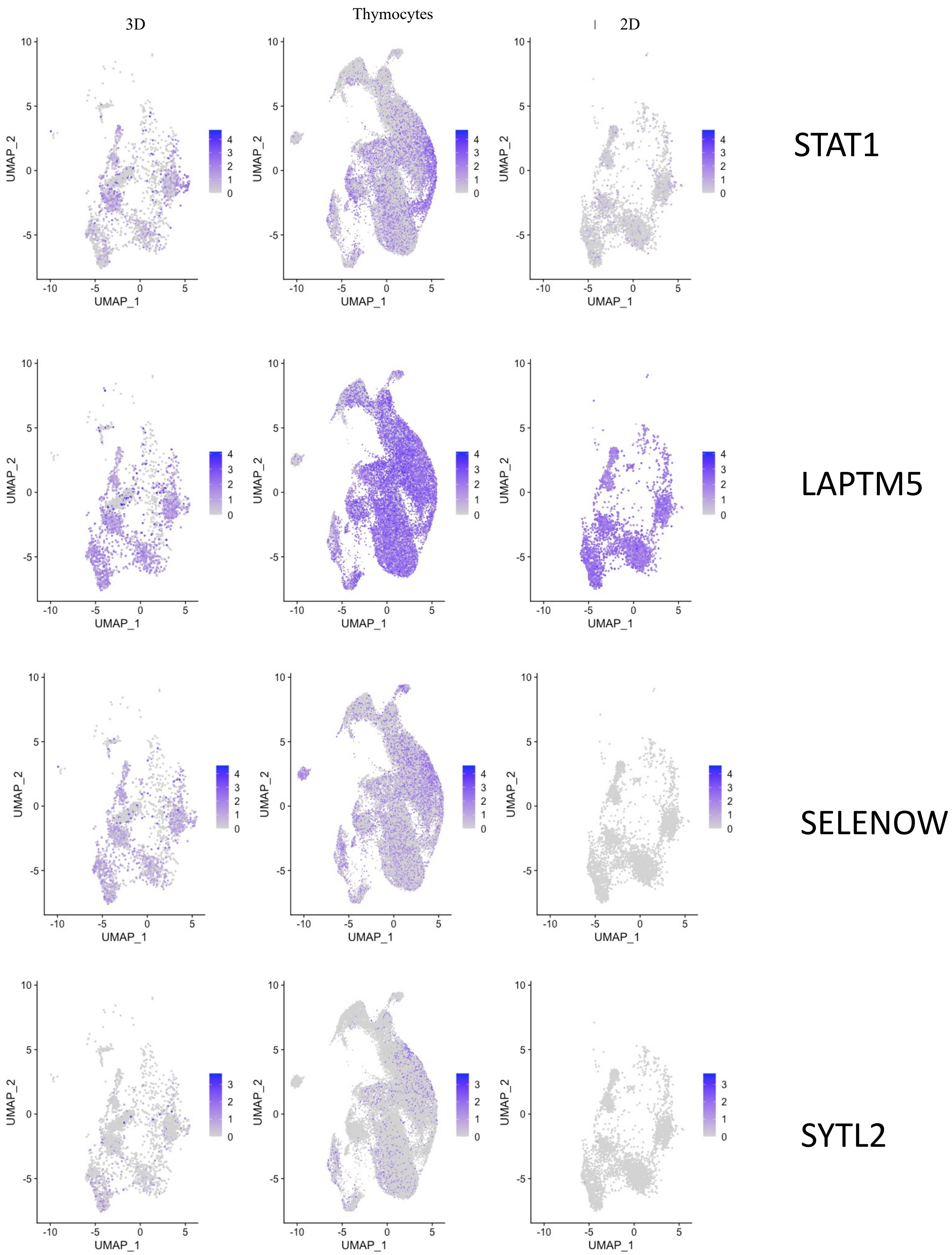
3D
Thymocytes
2D
STAT1
LAPTM5
SELENOW
SYTL2
UMAP_1
UMAP_2

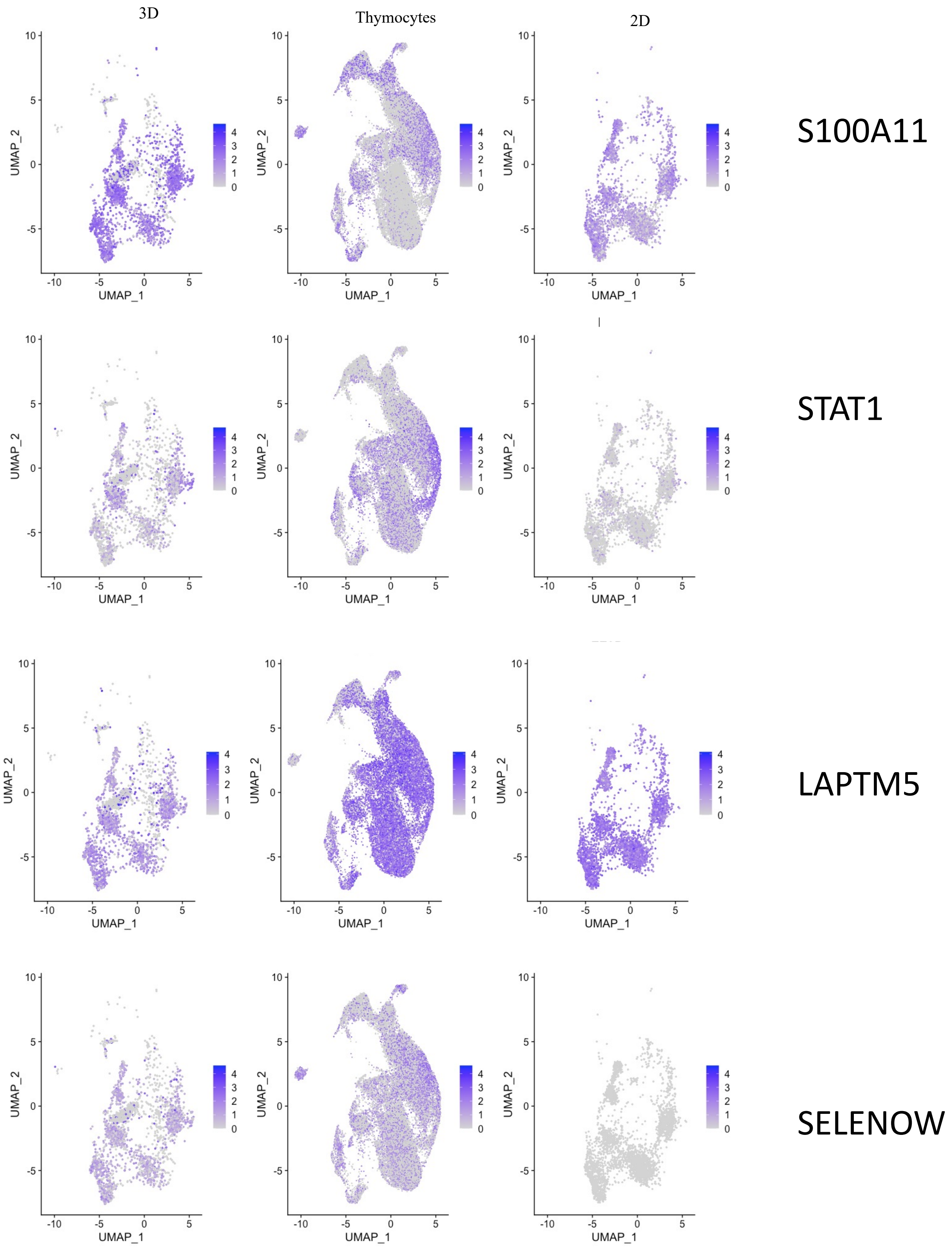
3D
Thymocytes
2D
S100A11
STAT1
LAPTM5
SELENOW
UMAP_1
UMAP_2

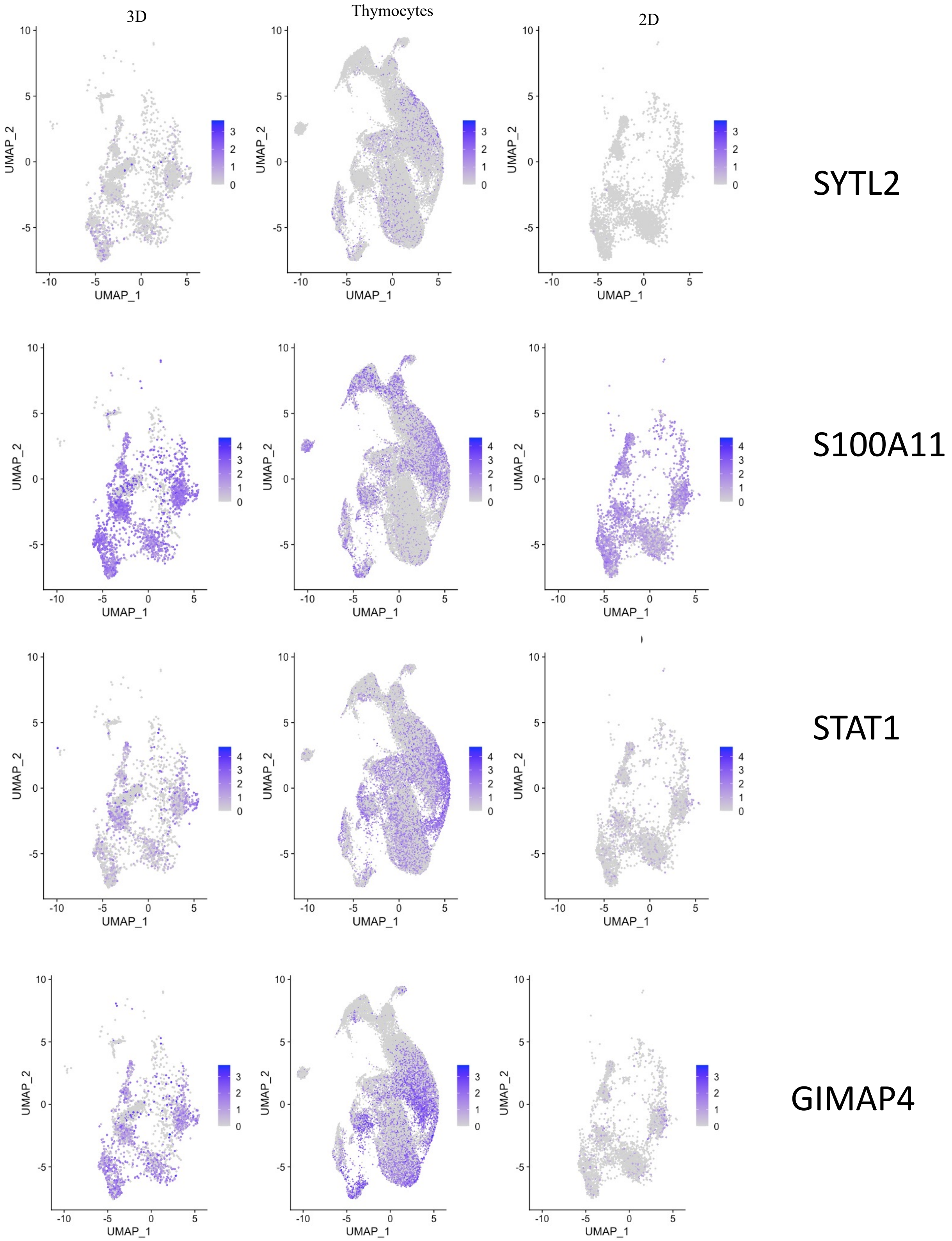

3D
Thymocytes
2D
SYTL2
S100A11
STAT1
GIMAP4
UMAP_1
UMAP_2

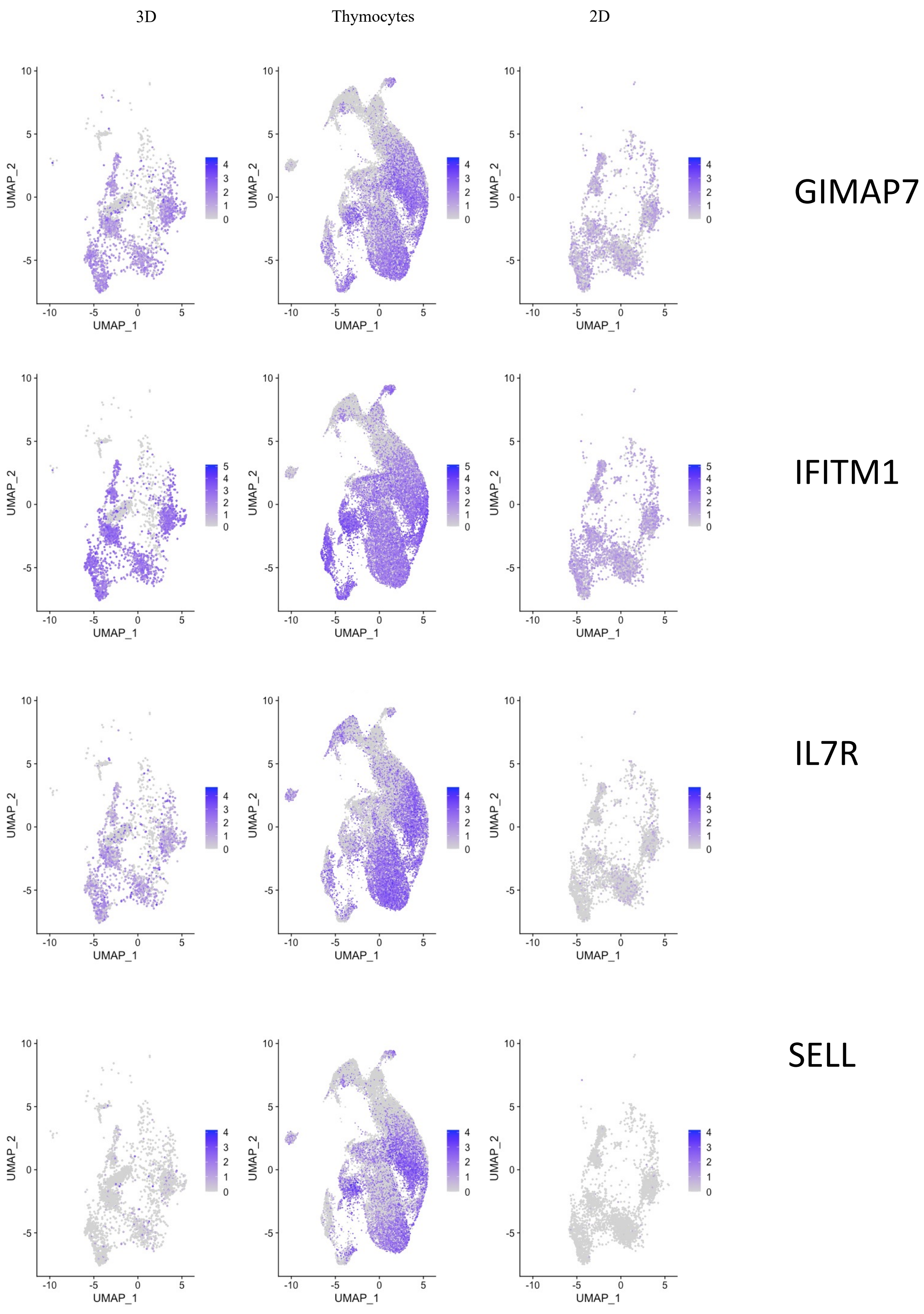
3D
Thymocytes
2D
GIMAP7
IFITM1
IL7R
SELL
UMAP_1
UMAP_2

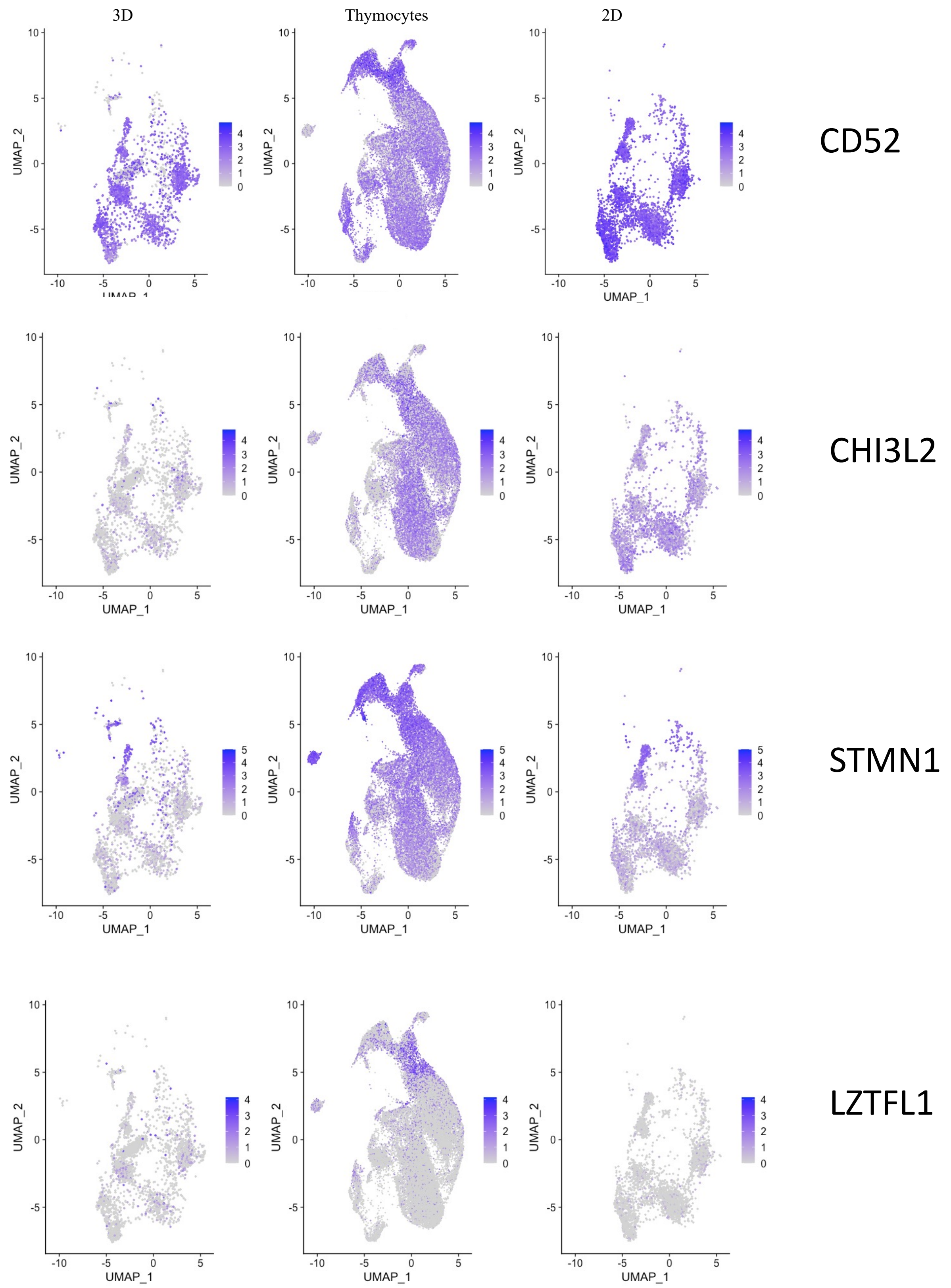
3D
Thymocytes
2D
CD52
CHI3L2
STMN1
LZTFL1
UMAP_1
UMAP_2

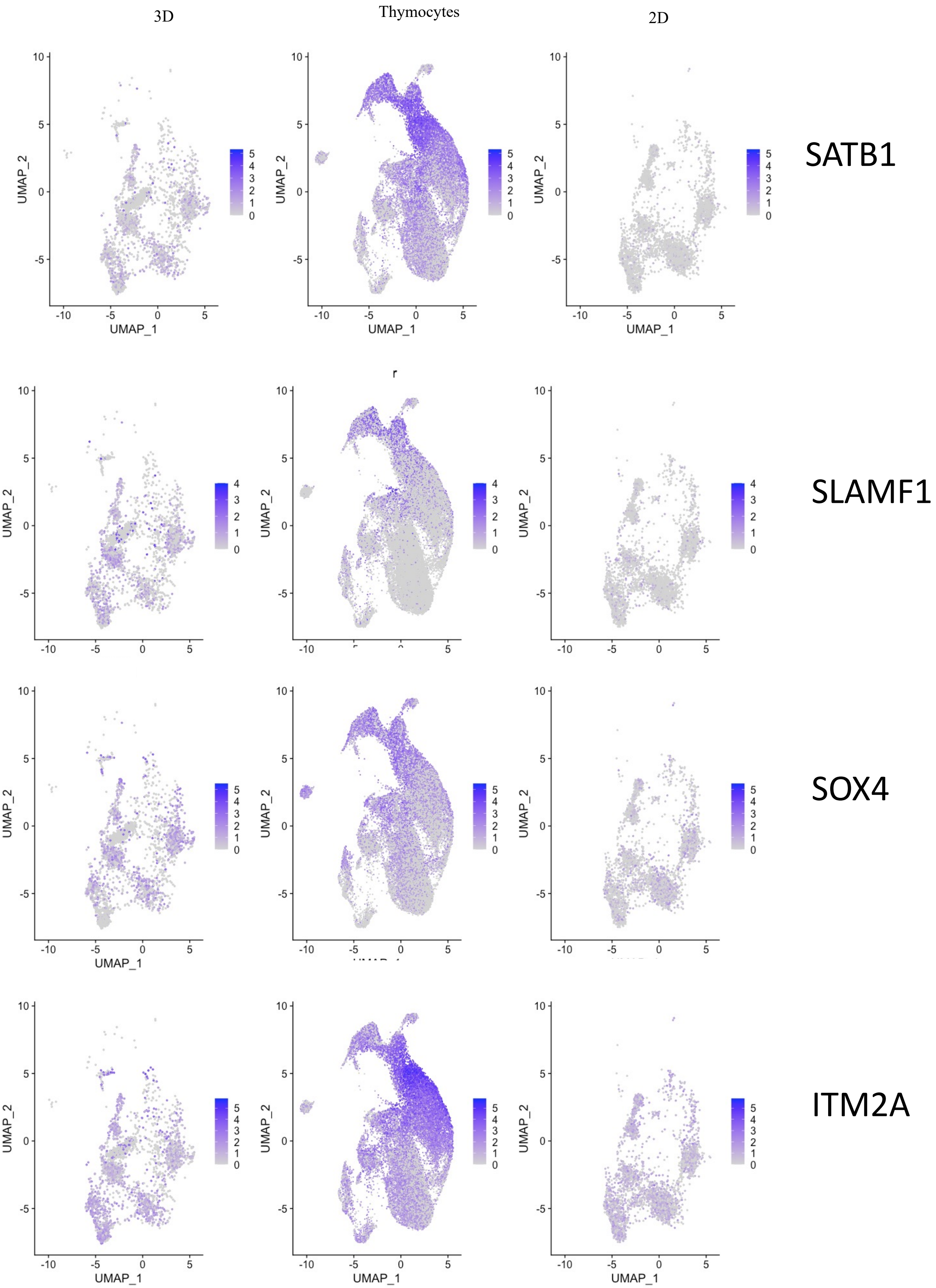
3D
Thymocytes
2D
SATB1
SLAMF1
SOX4
ITM2A
UMAP_1
UMAP_2

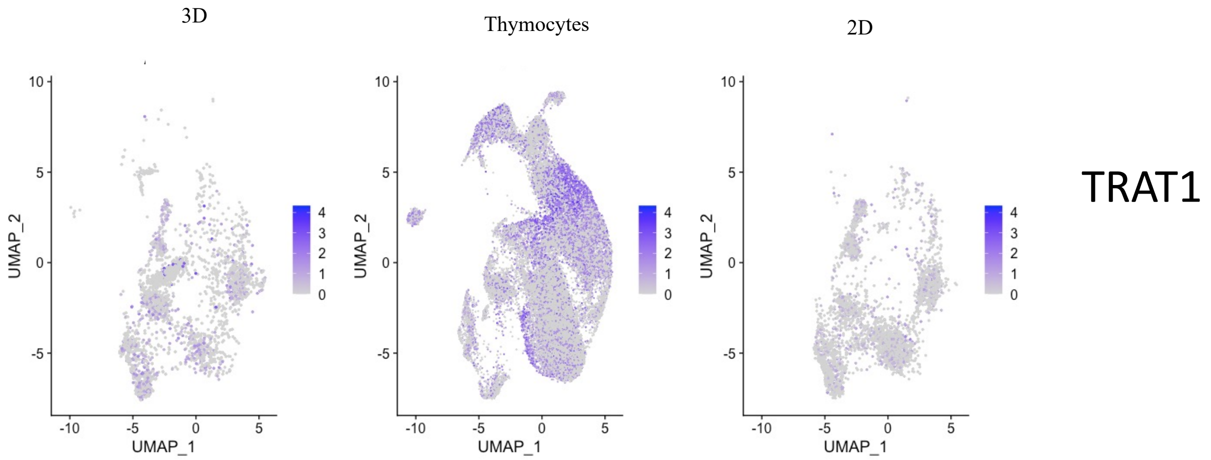
3D
Thymocytes
2D
UMAP_2
UMAP_1
TRAT1
